# Large-scale deep multi-layer analysis of Alzheimer’s disease brain reveals strong proteomic disease-related changes not observed at the RNA level

**DOI:** 10.1038/s41593-021-00999-y

**Published:** 2022-02-03

**Authors:** Erik C. B. Johnson, E. Kathleen Carter, Eric B. Dammer, Duc M. Duong, Ekaterina S. Gerasimov, Yue Liu, Jiaqi Liu, Ranjita Betarbet, Lingyan Ping, Luming Yin, Geidy E. Serrano, Thomas G. Beach, Junmin Peng, Philip L. De Jager, Vahram Haroutunian, Bin Zhang, Chris Gaiteri, David A. Bennett, Marla Gearing, Thomas S. Wingo, Aliza P. Wingo, James J. Lah, Allan I. Levey, Nicholas T. Seyfried

**Affiliations:** 1 Goizueta Alzheimer’s Disease Research Center, Emory University School of Medicine, Atlanta, GA USA; 2 Department of Neurology, Emory University School of Medicine, Atlanta, GA USA; 3 Department of Biochemistry, Emory University School of Medicine, Atlanta, GA USA; 4 Department of Genetics, Emory University School of Medicine, Atlanta, GA USA; 5 Banner Sun Health Research Institute, Sun City, AZ USA; 6 Departments of Structural Biology and Developmental Neurobiology, St. Jude Children’s Research Hospital, Memphis, TN USA; 7 Center for Proteomics and Metabolomics, St. Jude Children’s Research Hospital, Memphis, TN USA; 8 Center for Translational & Computational Neuroimmunology, Department of Neurology, Taub Institute, Columbia University Irving Medical Center, New York Presbyterian Hospital, New York, NY USA; 9 Departments of Psychiatry and Neuroscience, Icahn School of Medicine at Mount Sinai, New York, NY USA; 10 James J. Peters VA Medical Center MIRECC, Bronx, NY USA; 11 Department of Genetics and Genomic Sciences, Mount Sinai Center for Transformative Disease Modeling, Icahn School of Medicine at Mount Sinai, New York, NY USA; 12 Rush Alzheimer’s Disease Center, Rush University Medical Center, Chicago, IL USA; 13 Department of Pathology and Laboratory Medicine, Emory University School of Medicine, Atlanta, GA USA; 14 Department of Psychiatry, Emory University School of Medicine, Atlanta, GA USA; 15 Division of Mental Health, Atlanta VA Medical Center, Atlanta, GA USA

**Keywords:** Biochemical networks, Alzheimer's disease, Mass spectrometry, Alzheimer's disease, Proteomic analysis

## Abstract

The biological processes that are disrupted in the Alzheimer’s disease (AD) brain remain incompletely understood. In this study, we analyzed the proteomes of more than 1,000 brain tissues to reveal new AD-related protein co-expression modules that were highly preserved across cohorts and brain regions. Nearly half of the protein co-expression modules, including modules significantly altered in AD, were not observed in RNA networks from the same cohorts and brain regions, highlighting the proteopathic nature of AD. Two such AD-associated modules unique to the proteomic network included a module related to MAPK signaling and metabolism and a module related to the matrisome. The matrisome module was influenced by the *APOE ε4* allele but was not related to the rate of cognitive decline after adjustment for neuropathology. By contrast, the MAPK/metabolism module was strongly associated with the rate of cognitive decline. Disease-associated modules unique to the proteome are sources of promising therapeutic targets and biomarkers for AD.

## Abstract (web summary)

The authors analyzed the levels of more than 8,600 proteins across more than 1,000 brain tissues to arrive at a consensus AD brain protein co-expression network that illustrates the complexity and multiple pathological processes that occur in AD, many of which are not reflected at the RNA level.

## Main

AD remains a considerable personal and public health burden without effective disease-modifying therapies. As part of a national effort to develop therapeutics and biomarkers for AD, the Accelerated Medicines Partnership for Alzheimer’s Disease (AMP-AD) Consortium has been leveraging unbiased molecular profiling data at the genomic, transcriptomic, proteomic and metabolomic levels to further understanding of AD pathogenesis. Genetics has substantially advanced understanding of AD heritable risk, yet how genetic risk factors affect biological pathways that influence AD pathophysiology is not always clear^1^. Understanding the biological effects of AD risk factor polymorphisms in human brain often requires additional levels of analysis using other -omics approaches. To this end, transcriptomics has been widely used to measure mRNA transcripts in AD brain, and the resulting transcriptomic data have been integrated with AD genetic risk^2^. However, the ultimate biological effectors of AD genetic and environmental risk are often the proteins and the metabolic pathways they modulate. Compared to genomics and transcriptomics, proteomics approaches have, to date, provided comparatively less in-depth coverage of the target analyte due to increased complexity and technical demands of analyzing amino acid polymers versus nucleic acid polymers.

In this study, we used a tandem mass tag mass spectrometry (TMT-MS) approach^3–6^ and the AMP-AD Consortium of postmortem brain tissues to generate a deep TMT AD protein network that considerably expanded our previous label-free quantitation mass spectrometry (LFQ-MS) network^7^ and revealed new AD-related protein co-expression modules. We leveraged brain tissues from cohorts that also have been profiled using other -omics modalities, including genomics and transcriptomics, to perform a multi-layer genomic, transcriptomic and proteomic analysis of the TMT AD protein network to better understand the relationships among these different data types in the context of AD.

## Results

### TMT consensus AD protein co-expression network

**Fig. 1:**
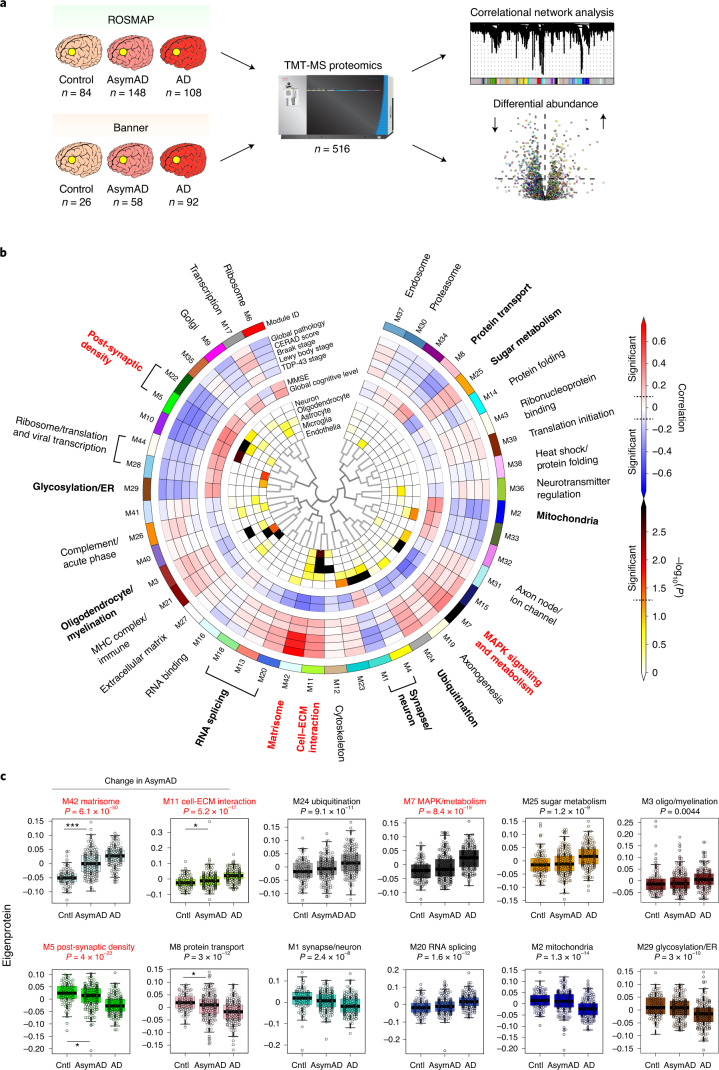
TMT AD protein co-expression network. **a–c**, 516 DLPFC tissues from the ROSMAP (*n* = 84 control, 148 AsymAD and 108 AD) and the Banner Sun Health Brain Bank (Banner, *n* = 26 control, 58 AsymAD and 92 AD) were analyzed by TMT-MS-based proteomics (**a**). After outlier removal and data processing, a total of 8,619 proteins were quantified across 488 cases, which were analyzed by both differential expression and co-expression approaches. **b**, A protein co-expression network was built using WGCNA, which consisted of 44 protein co-expression modules. Module relatedness is shown in the central dendrogram. GO analysis was used to identify the principal biology represented by each module. Modules that did not have a clear ontology were not assigned an ontology term. Module eigenproteins were correlated with neuropathological and cognitive traits present in the ROSMAP and Banner cohorts (red, positive correlation; blue, negative correlation). The global pathology, Lewy body stage, TDP-43 stage and global cognitive level traits were present only in ROSMAP. Twelve of the 44 modules that were most highly correlated to neuropathological and/or cognitive traits are in bold, with the four most strongly trait-related modules highlighted in red. The cell type nature of each module was assessed by module protein overlap with cell-type-specific marker lists of neurons, oligodendrocytes, astrocytes, microglia and endothelia. **c**, Module eigenprotein levels by case status for the 12 most strongly trait-correlated modules bolded in **b**. Modules are grouped by those that change in AsymAD (*n* = 4, left) and those that change only in AD (*n* = 8, right). *n* = 106 control, 200 AsymAD and 182 AD. Differences in module eigenprotein by case status were assessed by one-way ANOVA with Tukey test. *P < 0.05 and ***P < 0.001. Box plots represent the median and 25th and 75th percentiles, and box hinges represent the interquartile range of the two middle quartiles within a group. Data points up to 1.5 times the interquartile range from the box hinge define the extent of whiskers (error bars). Cntl, control; MHC, major histocompatibility complex.

**Extended Data Fig. 1:**
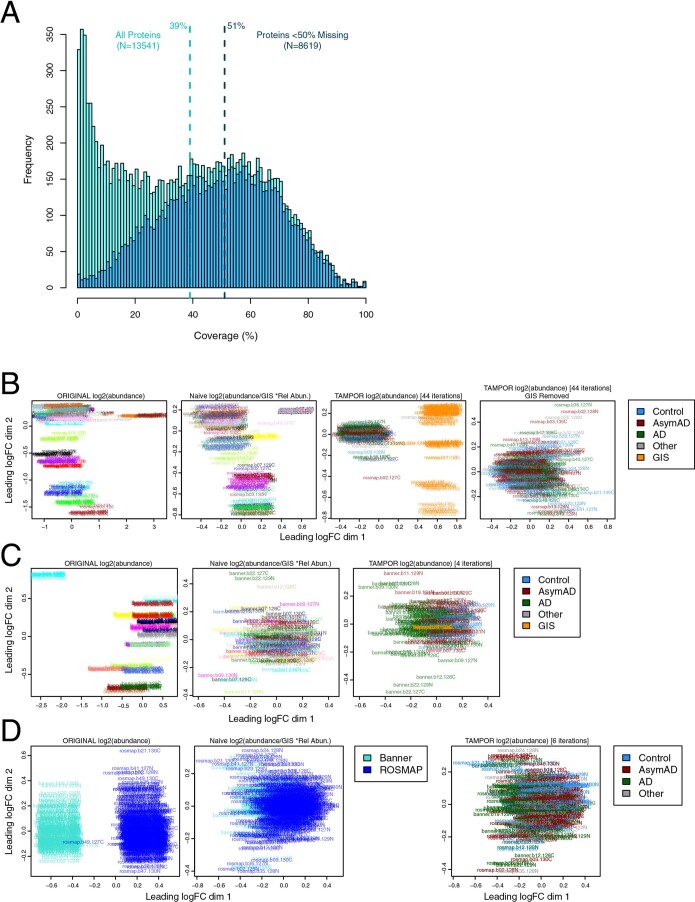
Percent Protein Coverage by TMT-MS and TMT-MS Batch Correction. (**a**) Percent protein coverage for all proteins measured (*n* = 13,541) and those measured in at least 50% of cases (*n* = 8,619), which was the threshold for inclusion in the co-expression network. The vertical dashed lines represent median percent coverage for each distribution. (**b–d**) TMT-MS batch correction as illustrated by multidimensional scaling (MDS). Starting log2 abundance, log2 abundance divided by the global internal standard (GIS), and additional batch correction by the TAMPOR algorithm in the ROSMAP cohort (**b**) and the Banner cohort (**c**) separately, followed by final cohort correction (**d**). Each batch is designated an arbitrary color in the first two panels in (**b**) and **(c**). dim, dimension; logFC, log fold change.

**Extended Data Fig. 2:**
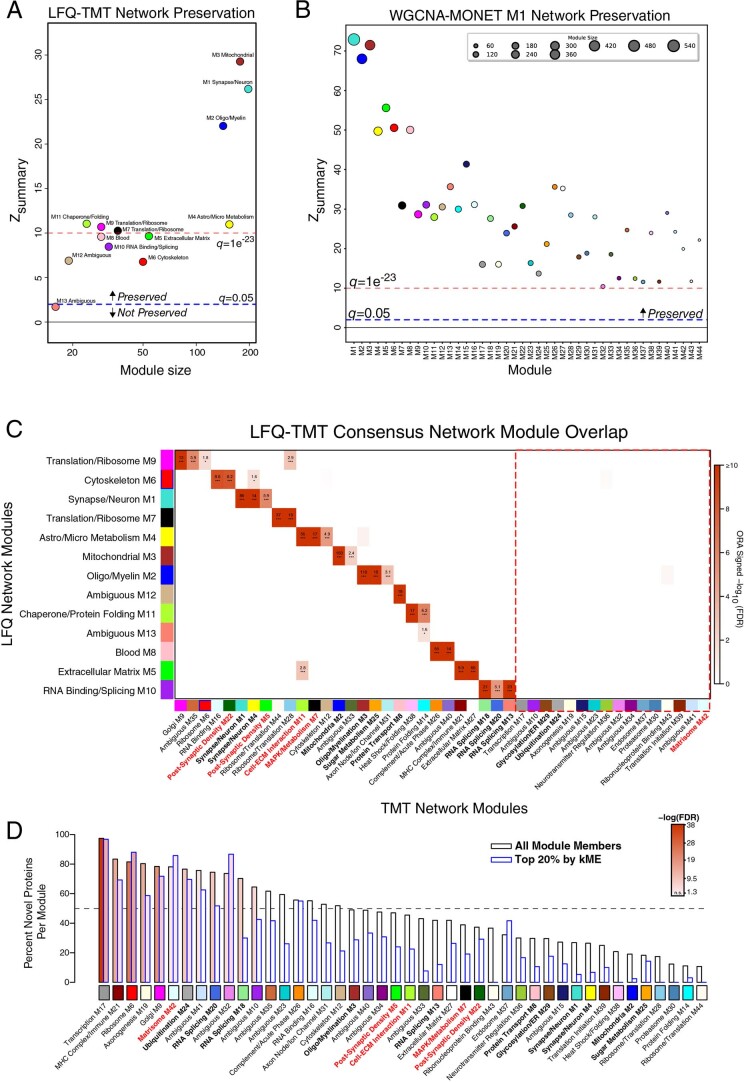
LFQ and TMT AD Network Comparison. (**a–d**) LFQ AD network module preservation in the TMT AD network (**a**). Modules that had a *Z*_summary_ score of greater than or equal to 1.96 (or *q* = 0.05, blue dotted line) were considered to be preserved, while modules that had a *Z*_summary_ score greater than or equal to 10 (or *q* = 1e^−23^, red dotted line) were considered to be highly preserved. (**b**) Preservation of the TMT AD network built using the weighted correlational network algorithm (WGCNA) into the network built on the same matrix using the MONET M1 algorithm. (**c**) Module member overrepresentation analysis (ORA) of the LFQ and TMT AD networks. The dashed red box highlights modules that are unique to the TMT network. The numbers in each box represent the –log_10_(FDR) value for the overlap. The heatmap is thresholded at a minimum of FDR(0.1) for clarity. (**d**) Percent novelty of TMT network module protein members compared to LFQ network proteins for all module members (black) or the top 20% of module proteins by strength of correlation to the module eigenprotein (kME) (blue). The dashed line indicates 50% novel protein members. Bars are shaded according to *P* value significance. ORA and percent novelty *P* values were corrected by the Benjamini-Hochberg procedure. * <0.05, ** <0.01, *** <0.005.

For this study, we analyzed a total of 516 dorsolateral prefrontal cortex (DLPFC) tissues from control, asymptomatic AD (AsymAD) and AD brains from the Religious Orders Study and Memory and Aging Project (ROSMAP, *n* = 84 control, 148 AsymAD and 108 AD)^8–10^ and the Banner Sun Health Research Institute (Banner, *n* = 26 control, 58 AsymAD and 92 AD)^11^ by TMT-MS-based quantitative proteomics (Fig. 1a and Supplementary Table 1). Cases were defined based on a unified classification scheme using semi-quantitative histopathological measures of Aβ and tau neurofibrillary tangle deposition^12–15^ as well as cognitive function near time of death, as previously described^7^. AsymAD cases were those with neuropathological burden of Aβ plaques and tau tangles similar to AD cases but without significant cognitive impairment near time of death, which is considered to be an early preclinical stage of AD^16^. After data processing and outlier removal, a total of 8,619 proteins were used to build a protein co-expression network using the weighted gene co-expression network analysis (WGCNA) algorithm^17^ (Fig. 1b, Extended Data Fig. 1 and Supplementary Tables 2–4). This network consisted of 44 modules or communities of proteins related to one another by their co-expression across control and disease tissues. Compared to our previous AD consensus network constructed using LFQ proteomic data, the TMT consensus network contained over five times as many proteins that could be assigned to a module (6,337 versus 1,205) as well as a larger fraction of quantified proteins that could be assigned to a module (73% versus 36%), highlighting the improved depth and coherence of the TMT data compared to the LFQ consensus data. Of the 13 modules previously identified in the LFQ consensus network^7^, every module except the smallest module (module 13 consisting of 20 proteins) was preserved in the TMT network (Extended Data Fig. 2a), also highlighting the consistency of the LFQ and TMT proteomic data. Because different network clustering algorithms can produce disparate networks, we tested the robustness of the TMT consensus network generated by the WGCNA algorithm by also generating a co-expression network using an independent algorithm—the MONET M1 algorithm. MONET M1 was identified as one of the top performers in the Disease Module Identification DREAM Challenge and is based on a modularity optimization algorithm rather than the hierarchical clustering approach used in WGCNA^18,19^. We found that all 44 WGCNA modules were highly preserved in the MONET M1 network (Extended Data Fig. 2b), demonstrating the robustness of the TMT consensus network to clustering algorithm.

The biology represented by each TMT consensus network module was determined using Gene Ontology (GO) analysis of its constituent proteins (Fig. 1b and Supplementary Data). Most modules could be assigned a primary ontology, and those that were ambiguous in their ontology were left unannotated or assigned as ‘ambiguous’. Module 42 was assigned the term ‘matrisome’, which refers to the collection of extracellular matrix (ECM)-associated proteins^20,21^, owing to its strong enrichment in ECM and glycosaminoglycan-binding proteins. To assess whether a module was related to features of AD, we correlated each module eigenprotein, or the first principal component (PC) of module protein expression, to neuropathological or cognitive traits present in the ROSMAP and Banner cohorts (Fig. 1b and Supplementary Table 5; individual protein trait correlations are provided in Supplementary Table 6). We also assessed the cell type nature of each module by determining whether it was enriched in cell-type-specific protein markers (Fig. 1b and Supplementary Tables 7 and 8). Because the network was highly powered, with the ability to observe a significant correlation of 0.1 at *P* = 0.05 for most pathological and cognitive traits, we were able to observe a large fraction of the 44 modules that significantly correlated with at least one pathological or cognitive trait. Twelve modules or module families were noted to correlate more strongly to AD traits than the others. These included post-synaptic density, glycosylation/endoplasmic reticulum (ER), oligodendrocyte/myelination, RNA splicing, matrisome, cell–ECM interaction, synapse/neuron, ubiquitination, mitogen-activated protein kinase (MAPK) signaling and metabolism, mitochondria, sugar metabolism and protein transport modules (Fig. 1b). Four of these modules—M5 post-synaptic density (global pathology *r* = –0.32, *P* = 2.7 × 10^−9^; global cognitive function *r* = 0.35, *P* = 7.4 × 10^−11^), M7 MAPK signaling and metabolism (global pathology *r* = 0.37, *P* = 4.9 × 10^−12^; global cognitive function *r* = –0.42, *P* = 1.2 × 10^−15^), M11 cell–ECM interaction (global pathology *r* = 0.34, *P* = 4.1 × 10^−10^; global cognitive function *r* = –0.33, *P* = 1.1 × 10^−9^) and M42 matrisome (global pathology *r* = 0.75, *P* = 1.1 × 10^−60^; global cognitive function *r* = –0.4, *P* = 2.3 × 10^−14^)—were the most strongly correlated to AD neuropathology or cognition out of the 12. The additional analytical depth afforded by the TMT pipeline also allowed us to identify a significant number of new modules that had little to no overlap with the LFQ network. Among these were the M17 transcription, M21 major histocompatibility complex/immune, M6 ribosome, M19 axonogenesis and M9 Golgi modules, in which approximately 80% or more of the module proteins were not quantified in the LFQ network, including a majority of proteins with strong correlation to the module eigenprotein (that is, ‘hub’ proteins) (Extended Data Fig. 2c,d). Three of these new modules—the M24 ubiquitination, M29 glycosylation/ER and M42 matrisome modules—were strongly correlated to AD endophenotypes.

To assess whether a given TMT network module was altered in the early stages of AD, we compared the module eigenprotein across control, AsymAD and AD cases (Fig. 1c, Supplementary Table 9 and Supplementary Data). Four of the 12 most highly AD-correlated modules were either significantly increased or significantly decreased in AsymAD compared to control, whereas the other eight were largely altered in AD only. Modules that were increased in AsymAD included M42 matrisome and M11 cell–ECM interaction, whereas modules that were decreased in AsymAD included M5 post-synaptic density and M8 protein transport.

Modules that correlated most strongly with elevated tau microtubule-binding domain (MTBR) peptide levels were the M42 matrisome and M11 cell–ECM modules, whereas modules that correlated most strongly in a negative direction with MTBR were M5 post-synpatic density and M1 and M4 synapse/neuron modules (Supplementary Tables 10–12). Modules that correlated with MTBR tended to be also altered in AsymAD.

In summary, TMT-MS analysis of more than 500 brain tissues allowed us to quantify more than 8,600 proteins and construct a robust protein co-expression network that was highly powered to detect AD-correlated modules, including a substantial number of new modules not present in the previous LFQ consensus network. Some of these new modules were also altered in early stages of AD, likely reflecting pathophysiologic processes that develop in the presence of AD neuropathology but before cognitive decline, and correlated with tau dyshomeostasis.

### Modules are preserved across cohorts and brain regions

**Fig. 2:**
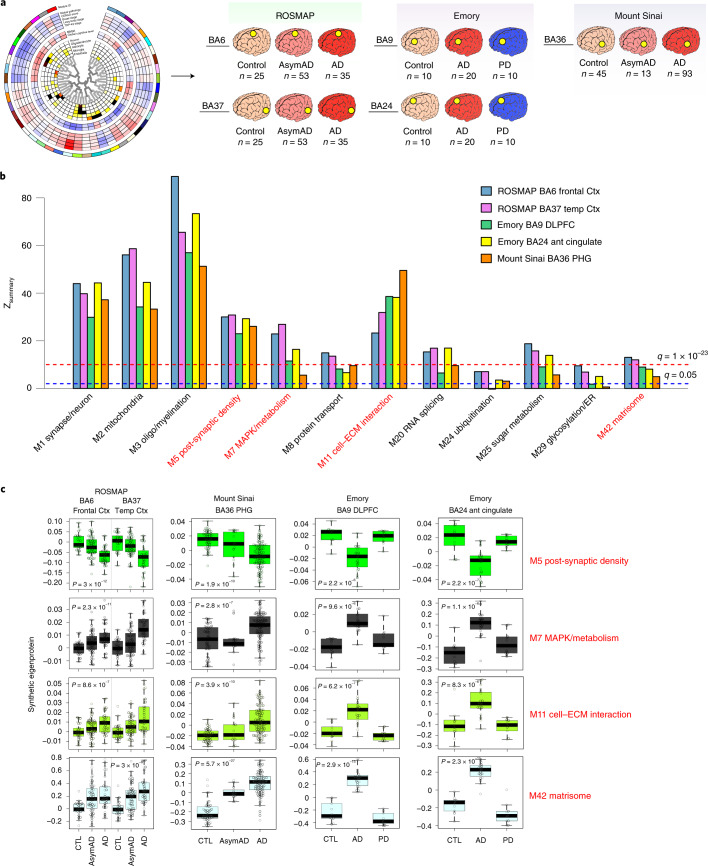
Preservation of the TMT AD network across different cohorts, centers, methods and brain regions. **a**–**c**, Module preservation and synthetic eigenprotein analysis of the TMT AD network generated from DLPFC BA9 tissues was performed in ROSMAP BA6 (frontal cortex) and BA37 (temporal cortex), Emory BA9 (DLPFC) and BA24 (anterior cingulate) and Mount Sinai Brain Bank BA36 PHG tissues (**a**). The Emory tissues included PD cases and were analyzed using a different TMT quantification approach (SPS-MS3). The Mount Sinai tissues were processed and analyzed by MS2-based TMT-MS at a different center. **b**, Module preservation of the 12 trait-correlated modules highlighted in Fig. 1b,c. Modules that had a *Z*_summary_ score greater than or equal to 1.96 (or *q* = 0.05, blue dotted line) were considered to be preserved, whereas modules that had a *Z*_summary_ score greater than or equal to 10 (or *q* = 1 × 10^−23^, red dotted line) were considered to be highly preserved. Preservation statistics for all TMT AD network modules are provided in Extended Data Fig. 3. **c**, Module eigenprotein level by case status was assessed in the different cohorts and brain regions shown in **a** by measuring a TMT AD network synthetic eigenprotein, representing the top 20% of module proteins by module kME, in each cohort and region. Synthetic eigenprotein levels are shown for the four most highly trait-correlated TMT AD network modules. Differences and statistics for all modules are provided in Supplementary Table 17. ROSMAP BA6 *n* = 25 control, 53 AsymAD, 35 AD; ROSMAP BA37 *n* = 25 control, 53 AsymAD, 35 AD; Emory BA9 *n* = 10 control, 20 AD, 10 PD; Emory BA24 *n* = 10 control, 20 AD, 10 PD; Mount Sinai BA36 *n* = 45 control, 13 AsymAD, 93 AD. Differences in synthetic eigenprotein levels were assessed by one-way ANOVA. Box plots represent the median and 25th and 75th percentiles, and box hinges represent the interquartile range of the two middle quartiles within a group. Data points up to 1.5 times the interquartile range from the box hinge define the extent of whiskers (error bars). ant, anterior; CTL, control; Ctx, cortex, temp, temporal.

**Extended Data Fig. 3:**
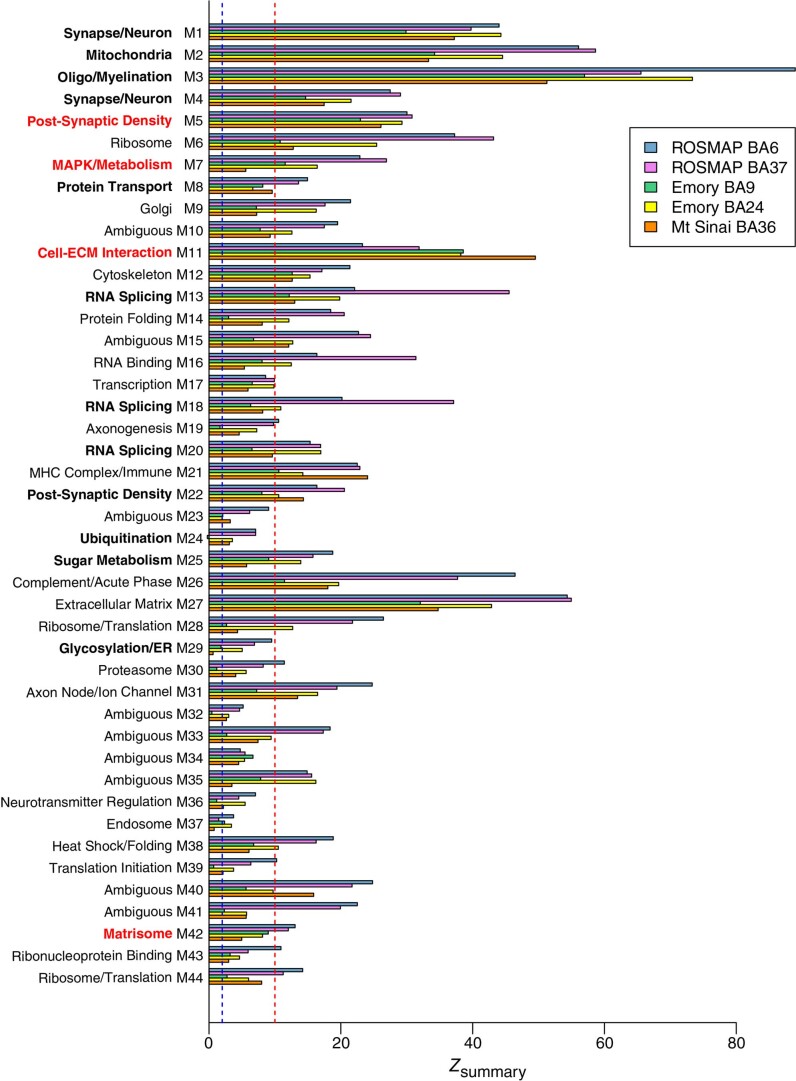
TMT AD Network Module Preservation. Modules that had a *Z*_summary_ score of greater than or equal to 1.96 (or *q* = 0.05, blue dotted line) were considered to be preserved, while modules that had a *Z*_summary_ score greater than or equal to 10 (or *q* = 1e^−23^, red dotted line) were considered to be highly preserved. AD, Alzheimer’s disease; Aβ, amyloid-β; AsymAD, asymptomatic Alzheimer’s disease; BA, Brodmann area; ECM, extracellular matrix; ER, endoplasmic reticulum; MAPK, mitogen-activated protein kinase.

The TMT AD protein network was generated from DLPFC Brodmann area 9 (BA9) tissues from two centers analyzed at one institution. To determine whether the network modules were also present in other brain regions and robust to center and analytical pipeline, we analyzed 226 paired tissues from frontal cortex BA6 (*n* = 113) and temporal cortex BA37 (*n* = 113) from 113 participants in the ROSMAP cohort, 151 parahippocampal gyrus (PHG) BA36 tissues from the Mount Sinai Brain Bank and 40 tissues from DLPFC BA9 and anterior cingulate BA24 from the Emory Brain Bank, which also included Parkinson’s disease (PD) cases (Fig. 2a and Supplementary Tables 13–16). The Mount Sinai tissues were analyzed at a different center using a similar mass spectrometry pipeline^22^. All tissues were analyzed using the TMT approach, with the Emory tissues analyzed using the synchronous precursor selection (SPS)-MS3 TMT quantification approach^3,23^. We generated protein co-expression networks for each cohort and then assessed whether the TMT AD network modules were preserved in each cohort and brain region^7,24^. We found that nearly all TMT AD network modules were preserved across both cohort and brain region (Fig. 2b and Extended Data Fig. 3).

We assessed how TMT AD network modules were different by case status in each cohort and brain region by measuring TMT consensus AD network ‘synthetic’ eigenproteins’, or the top 20% of proteins within each consensus module, in each separate network (Fig. 2c and Supplementary Table 17)^7^. Because the ROSMAP BA6 and BA37 tissues were sampled within subject, we were able to compare the module synthetic eigenproteins directly between these two regions within the same individual. All TMT AD network modules were altered in a similar direction to that observed in DLPFC across brain region and cohort. Interestingly, the M7 MAPK/metabolism and M42 matrisome modules were increased more strongly in temporal cortex than frontal cortex when assessed in the same individual, perhaps due to earlier and more severe involvement of this brain region in AD^25^. Most AD-associated modules were not significantly altered in PD in either frontal cortex or anterior cingulate, although there appeared to be a trend for the M7 MAPK/metabolism module to increase and the M5 post-synaptic density module to decrease in anterior cingulate, consistent with this brain region being more severely affected in PD compared to DLPFC^26,27^. In summary, we observed that nearly all TMT AD network modules were preserved across different cohorts, centers, MS methods and brain regions, demonstrating that the protein co-expression relationships observed are robust to technical artifact and are not unique to the DLPFC.

### Modules not observed in transcriptomic networks

**Fig. 3:**
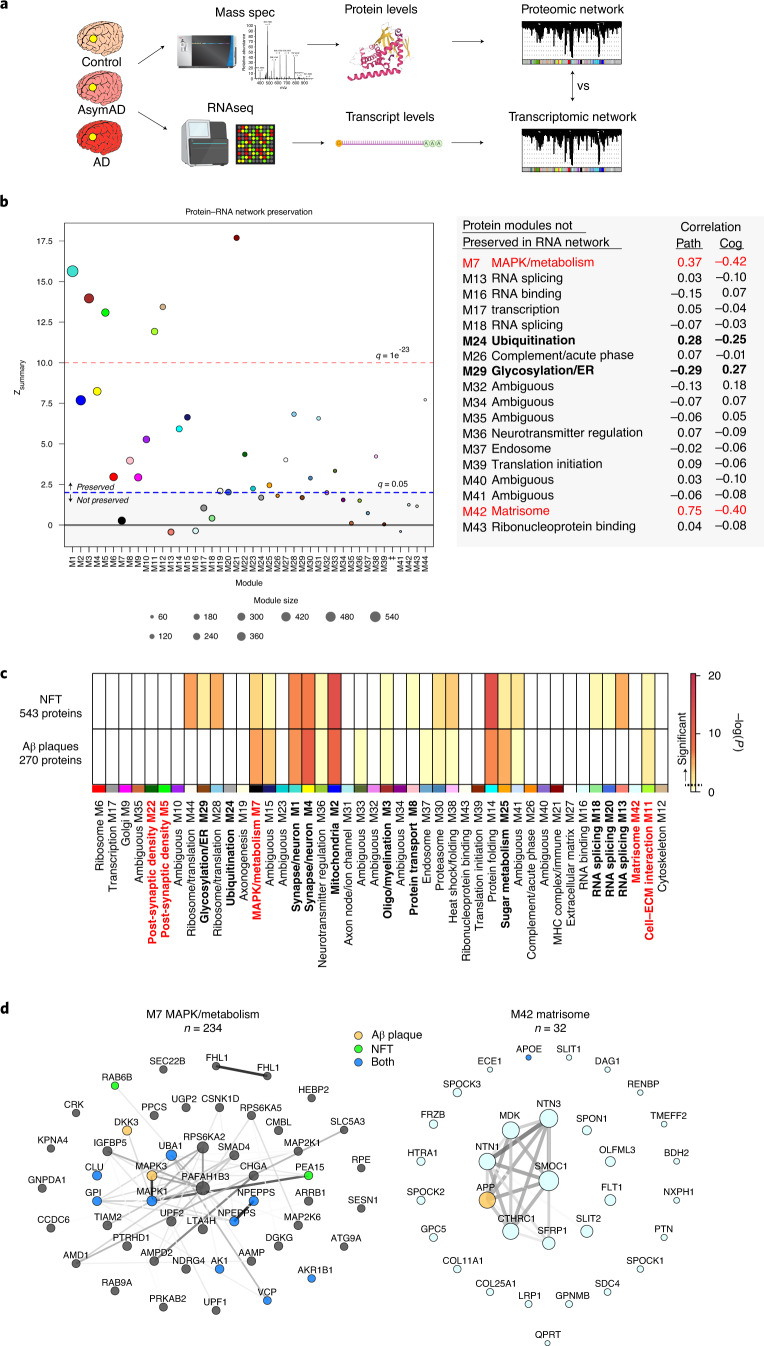
The TMT AD protein network contains modules associated with AD that are not present in the transcriptome. **a**–**d**, Control, AsymAD and AD frontal cortex tissues from both the ROSMAP cohort (BA9; control = 125, AsymAD = 204, AD = 203; 168 overlapping cases with proteomic analysis) and the Mount Sinai Brain Bank (BA10; control = 54, AsymAD = 19, AD = 120) were analyzed by RNA-seq-based transcriptomics and co-expression networks generated by WGCNA in similar fashion to the TMT AD protein network (**a**). **b**, Module preservation of the TMT AD protein network into the ROSMAP RNA network. Modules that had a preservation *Z*_summary_ score less than 1.96 (*q* > 0.05) were not considered to be preserved. Modules that had a *Z*_summary_ score greater than or equal to 1.96 (or *q* = 0.05, blue dotted line) were considered to be preserved, whereas modules that had a *Z*_summary_ score greater than or equal to 10 (or *q* = 1 × 10^−23^, red dotted line) were considered to be highly preserved. TMT AD network modules that were not preserved in the RNA network, along with their correlation to global pathology and global cognition traits in ROSMAP, are listed on the right. Additional information on modules preserved in ROSMAP, as well as preservation analysis with the Mount Sinai cohort, are provided in Extended Data Fig. 5a,b. **c**, TMT AD network module protein overlap with proteins identified as co-localized with NFTs (*n* = 543) and Aβ plaques (*n* = 270) as described by Drummond et al.^30,31^. Overlap as shown with a dark yellow hue or darker is considered significant. Overlap with a less stringent set of Aβ plaque-associated proteins is provided in Extended Data Fig. 4c. **d**, The top 50 proteins by module kME for the M7 MAPK/metabolism (left, *n* = 234 total proteins) and M42 matrisome (right, *n* = 32 total proteins) modules. Module proteins that were found to be co-localized with NFTs (green), Aβ plaques (orange) or both (blue) are highlighted. Lines between proteins represent correlation matrix adjacency weights. Graphs for all TMT AD network modules are provided in Supplementary Data. Cog, cognition; Path, pathology.

**Extended Data Fig. 4:**
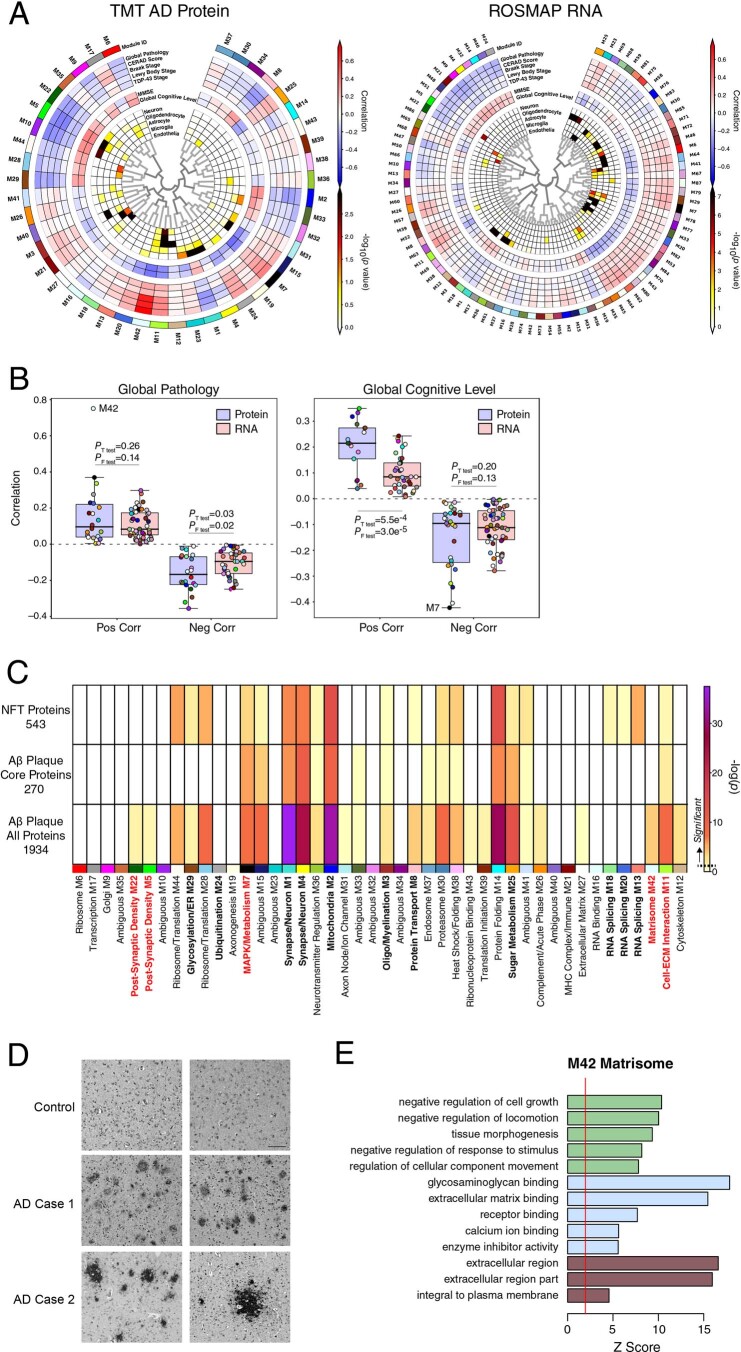
Protein and RNA AD Network Trait Correlations and TMT AD Network Module Overlap with Neurofibrillary Tangle and Aβ Plaque Proteins. (**a-b**) Module trait correlation analysis between protein and RNA networks. (**a**) WGCNA networks of TMT AD protein (left) and ROSMAP RNA (right). Protein and RNA data were obtained from the same brain region (dorsolateral prefrontal cortex, Brodmann area 9), with 168 ROSMAP cases shared between networks. Module eigenprotein to trait correlations are shown by red and blue heatmap. (**b**) Protein and RNA network module correlations with global pathology (left; *n* = 22 positive protein, 22 negative protein, 50 positive RNA, 38 negative RNA) and global cognitive level (right; n = 17 positive protein, 27 negative protein, 35 positive RNA, 53 negative RNA) as measured in ROSMAP. Differences in overall positive and negative correlations between protein and RNA modules were assessed by two-sided Welch’s *t* test, whereas differences in overall variation in correlation were measured by *F* test. *P* values for each test are provided. Boxplots represent the median, 25^th^, and 75^th^ percentiles, and box hinges represent the interquartile range of the two middle quartiles within a group. Datapoints up to 1.5 times the interquartile range from box hinge define the extent of whiskers (error bars). (**c**) TMT AD network module protein overlap with proteins identified as co-localized with neurofibrillary tangles (NFTs, *n* = 543) and amyloid-β (Aβ) plaques as described by Drummond *et al*.^30,31^. Overlap with Aβ plaques was performed with a set of proteins consistently observed in Aβ plaques across multiple experiments (Aβ plaque core proteins, *n* = 270), as well as with a set of proteins that included proteins observed only once across multiple experiments (Aβ plaque all proteins, *n* = 1934). Overlap was performed with one-sided Fisher’s exact test, and corrected by the Benjamini-Hochberg procedure. (**d**) Immunohistochemistry of midkine (MDK), a hub protein of the M42 matrisome module, in control and AD brain. Two out of three independent experiments are shown as representative. Scale bar represents 500 µM. (**E**) Gene ontology analysis of the M42 matrisome module, including biological process (green), molecular function (blue), and cellular component (brown) ontologies. The red line indicates a *z* score of 1.96, or *p* = 0.05.

**Extended Data Fig. 5:**
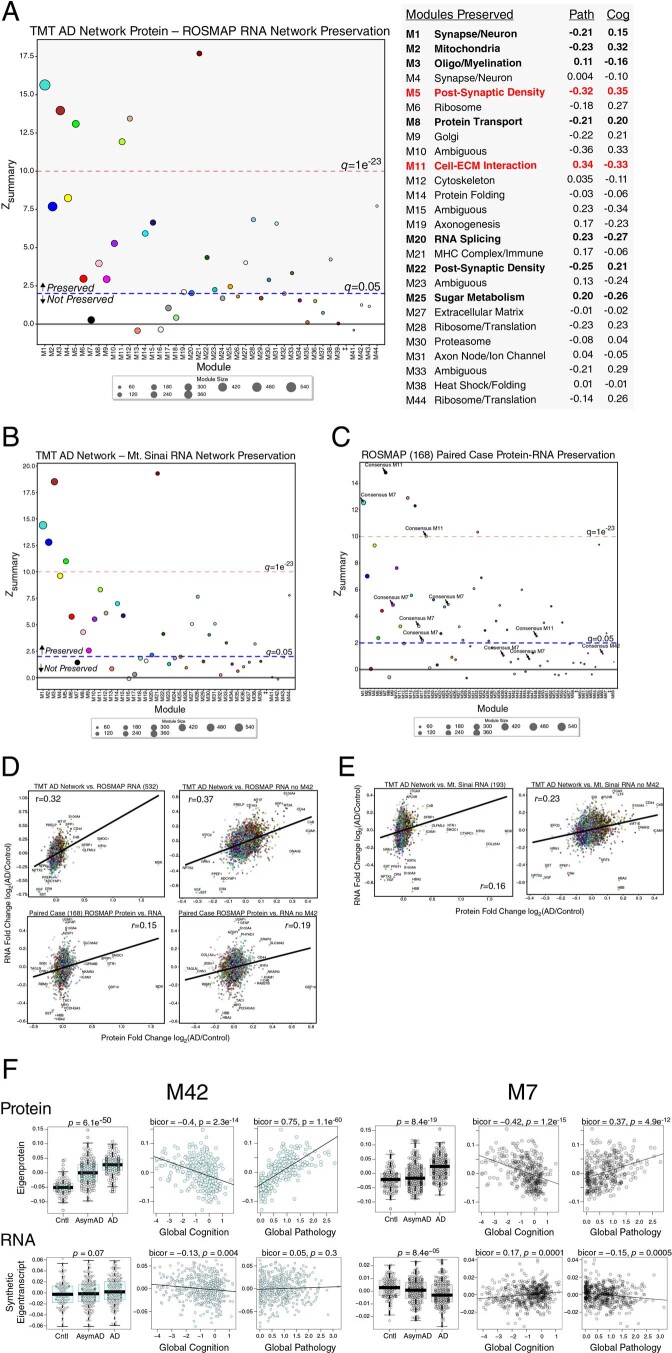
TMT AD Network Module Preservation in RNA Networks. (**a–f**) Module preservation of the TMT AD protein network into the ROSMAP RNA network (A). Modules that had a preservation *Z*_summary_ score less than 1.96 (*q* > 0.05) were not considered to be preserved. Modules that had a *Z*_summary_ score of greater than or equal to 1.96 (or *q* = 0.05, blue dotted line) were considered to be preserved, while modules that had a *Z*_summary_ score greater than or equal to 10 (or *q* = 1e^−23^, red dotted line) were considered to be highly preserved. TMT AD network modules that were preserved in the RNA network, along with their correlation to global pathology and global cognition traits in ROSMAP, are listed on the right. (**b**) Module preservation of the TMT AD protein network into the Mt. Sinai RNA network. (**c**) Module preservation of the ROSMAP 168 protein network into the paired case RNA network. AD protein network module assignments are provided for M7, M11, and M42. Additional module assignments are provided in Supplementary Table 21. (**d**) Correlation of AD *versus* control RNA and protein levels between the TMT protein and ROSMAP RNA (*n* = 532 cases) networks (top), as well as between cases paired between protein and RNA in ROSMAP (*n* = 168), including (left) or excluding (right) M42 proteins. (E) Correlation of AD *versus* control RNA and protein levels between the TMT protein and Mt. Sinai RNA (*n* = 193 cases) networks, including (left) or excluding (right) M42 proteins. Correlations were performed using Pearson correlation. (**f**) Comparison of M42 matrisome (left) and M7 MAPK/metabolism (right) eigenprotein (top; *n* = 106 control, 200 AsymAD, 182 AD, Total=488) and synthetic eigentranscript (bottom; *n* = 125 control, 204 AsymAD, 203 AD, Total=532) in ROSMAP cases levels by case status, and correlation with global pathology (*n* = 328 eigenprotein, 532 eigentranscript) and global cognitive function (*n* = 328 eigenprotein, 529 eigentranscript) in ROSMAP. Differences were assessed by one-way ANOVA. Correlations were performed using bicor. Boxplots represent the median, 25^th^, and 75^th^ percentiles, and box hinges represent the interquartile range of the two middle quartiles within a group. Datapoints up to 1.5 times the interquartile range from box hinge define the extent of whiskers (error bars).

**Extended Data Fig. 6:**
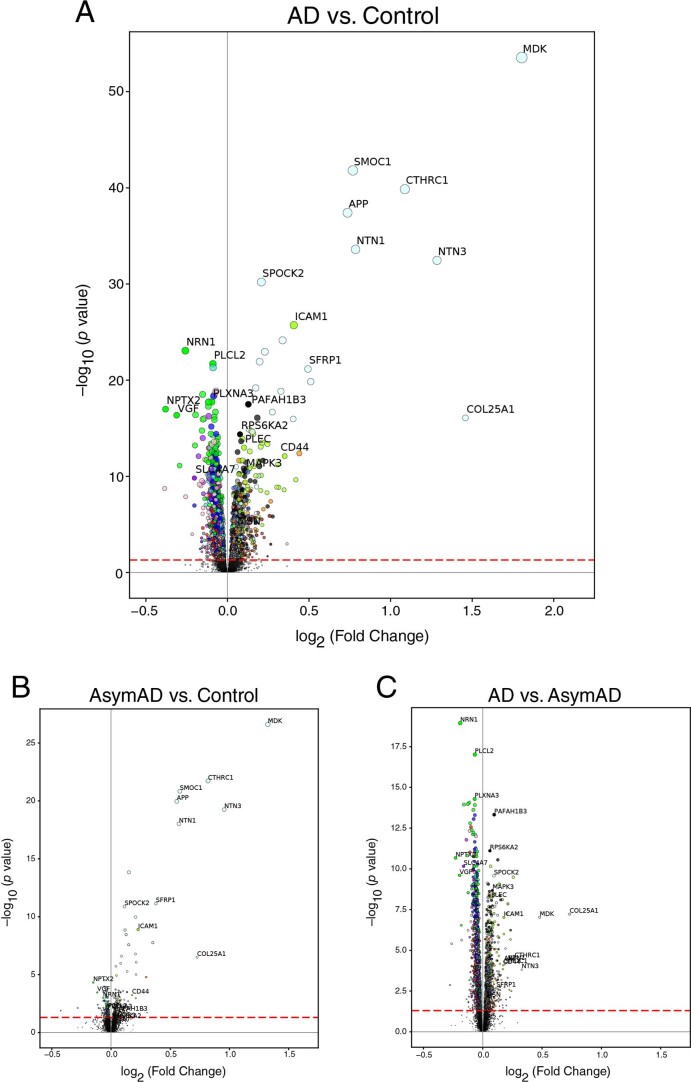
TMT AD Network Protein Differential Expression. (**a–c**) Differential expression between AD *versus* control (**a**), AsymAD *versus* control (B), and AsymAD *versus* AD (**c**). The dashed red line indicates a *P* value of 0.05, above which proteins are considered significantly differentially expressed. Proteins are colored by the network module in which they reside, according to the module color scheme provided in Fig. 1b. Proteins in the M42 matrisome module are colored lightcyan. One-way ANOVA followed by two-tailed pairwise test was used to calculate *P* values. Significance was adjusted by the Holm procedure. Fold change and statistics for all proteins are provided in Supplementary Table 2.

Most co-expression network analysis in AD has been performed to date using quantitative RNA sequencing (RNA-seq) data. However, not all mRNA transcripts correlate well with protein levels^28,29^. To compare the similarities and differences between RNA and protein AD co-expression networks, we generated an AD RNA network on 15,582 transcripts measured across 532 ROSMAP DLPFC tissues, 168 of which overlapped with tissues used to generate the TMT AD protein network (Fig. 3a and Supplementary Tables 18–20). We took care to ensure that the case classification and WGCNA pipeline used for network construction was consistent between protein and RNA datasets. Given the greater number of transcripts measured compared to proteins measured (*n* = 15,582 versus 8619), the resulting RNA network contained more modules than the protein network (n = 88 versus 44) (Extended Data Fig. 4a and Supplementary Table 20). We used network preservation statistics to determine which modules in the protein network were preserved in the RNA network^24^. We found that slightly more than half of the protein modules were preserved in the RNA network (26 of 44 modules at *Z*_summary_ > 1.96 or *P* ≤ 0.05) (Extended Data Fig. 5a). Among the modules preserved in the RNA network were the AD-associated modules M1 synapse/neuron, M2 mitochondria, M3 oligo/myelination, M5 and M22 post-synaptic density, M8 protein transport, M11 cell–ECM interaction, M20 RNA splicing and M25 sugar metabolism. However, there were also 18 protein network modules that were not preserved in the RNA network, including the AD-associated modules M7 MAPK/metabolism, M24 ubiquitination, M29 glycosylation/ER and M42 matrisome (Fig. 3b). Of these modules, the M7 MAPK/metabolism module—the module most highly correlated to cognitive function in the TMT AD network—was the least preserved, with a Z_summary_ score near 0, indicating its highly unique nature to the proteome. We validated these findings in 193 frontal cortex (BA10) tissues analyzed by RNA-seq from the Mount Sinai Brain Bank, which showed similar network preservation results (Extended Data Fig. 5b). We also analyzed separately the 168 ROSMAP cases that had paired proteomic and transcriptomic data from the DLPFC region (Extended Data Fig. 5c and Supplementary Tables 18 and 21). Approximately half (55%) of protein modules from this network were preserved in RNA, indicating that significant differences between protein and RNA co-expression exist even in tissue sampled from the same brain region in the same individual. Co-expression analysis indicated a degree of preservation between protein and RNA networks that was higher than what might be expected based on comparison of differential expression between protein and RNA, which was modest, even in paired tissues (Extended Data Fig. 5d,e). This correlation remained modest when proteins from the M42 matrisome module, which were the most highly differentially expressed proteins in the TMT AD network (Extended Data Fig. 6), were excluded from the analysis (Extended Data Fig. 5d,e). Overall, we found that protein network modules were correlated more strongly to cognitive function than RNA network modules, but that, in most cases, their correlation to pathology was similar to RNA modules (~0.15 on average in both positive and negative directions). A striking exception was the M42 matrisome module, which was the module with the strongest correlation to any AD trait, with correlation of 0.75 to global pathology, and which was not present in the RNA network (Extended Data Fig. 4b). A synthetic eigentranscript of M42 in the RNA network showed minimal relationship to disease, and some eigentranscripts—for instance, M7 MAPK/metabolism—demonstrated an opposite relationship to AD compared to protein, perhaps suggesting the presence of compensatory regulatory mechanisms at the RNA level for these module proteins (Extended Data Fig. 5f). The protein network also demonstrated an overall larger variance in module AD trait correlations than the RNA network. In summary, we observed that approximately half of the TMT AD protein network modules were not present in RNA networks from the same brain region, including the two protein network modules most strongly correlated to AD pathology and cognitive function, highlighting the unique contribution of the proteome to understanding AD pathophysiology.

### Modules enriched in proteins co-localized with plaques and tangles

To better understand the potential spatial relationships between the TMT AD protein network modules and the hallmark AD neuropathologies amyloid-β (Aβ) plaques and neurofibrillary tangles (NFTs), we performed a module overlap test with proteins that have previously been identified as co-localized with Aβ plaques and NFTs based on laser capture microdissection (LCM) and LFQ proteomic analysis of these structures (Fig. 3c and Supplementary Table 22)^30,31^. We found that the M1 and M4 synapse/neuron, M2 mitochondria and M14 protein folding modules were highly enriched in proteins found in both plaques and tangles. The M7 MAPK/metabolism module was also enriched in both plaques and tangles but more highly enriched in Aβ plaque-associated proteins. This was also the case for the M25 sugar metabolism module. NFTs were uniquely enriched in proteins from the M28 and M44 ribosome/translation, M29 glycosylation/ER and M13 RNA splicing modules. Surprisingly, the M42 matrisome module was not significantly enriched with core plaque proteins identified by LCM, even though the amyloid precursor protein (APP) (a proteomic measurement largely driven by Aβ) and apolipoprotein E (ApoE) were members of this module (Supplementary Table 4). M42 was highly elevated in AsymAD and AD compared to control, consistent with an association with neuritic plaques. When the analysis was expanded to a less stringent set of plaque-associated proteins identified in at least one LCM experiment rather than proteins identified in multiple experiments, M42 was found to be significantly enriched in plaque-associated proteins (Extended Data Fig. 4c). This suggested that our TMT proteomic and co-expression analysis was perhaps capturing a significant number of plaque-associated proteins that are less reliably observed by LFQ-MS approaches, even with LCM isolation, such as SPARC-related modular calcium-binding protein 1 (SMOC1), midkine (MDK) and netrin-1 (NTN1). For example, although it was not identified as a core Aβ plaque-associated protein by LCM, MDK demonstrated a pattern of staining on immunohistochemistry consistent with its co-localization with Aβ plaques (Extended Data Fig. 4d). Other proteins within the M7 MAPK/metabolism and M42 matrisome modules have been shown to co-localize with Aβ plaques and NFTs by immunohistochemistry^22,32–38^. Many of the proteins within the M42 matrisome module shared heparan sulfate and glycosaminoglycan-binding domains, likely mediating their interaction with Aβ fibrils^22^ (Extended Data Fig. 4e). NFT and core Aβ plaque proteins that overlap with the top 50 M7 MAPK/metabolism and M42 matrisome module proteins by module eigenprotein correlation value (kME) are shown in Fig. 3d. In summary, we found that several TMT AD protein network modules were enriched in proteins that are found in NFTs and Aβ plaques, including the M7 MAPK/metabolism module, consistent with a spatial relationship between these biological processes and hallmark AD pathologies.

### Matrisome module protein levels are influenced by APOE ε4

**Fig. 4:**
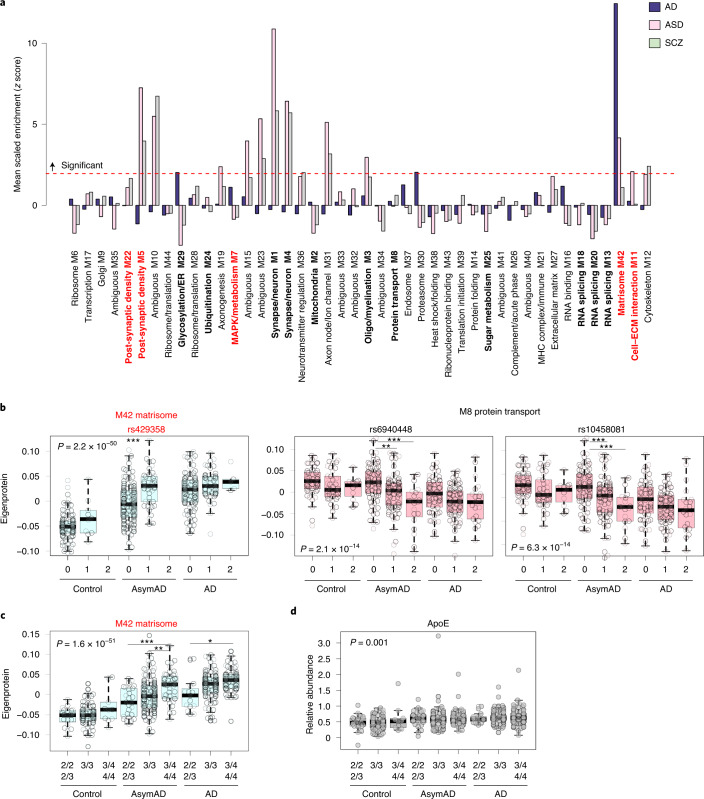
The M42 matrisome module is enriched in AD genetic risk and is increased by *APOE ε4*. **a**, **b**, Enrichment of AD genetic risk factor proteins as identified by GWAS in TMT AD network modules (**a**). The dashed red line indicates a *Z* score of 1.96 (*P* = 0.05), above which enrichment was considered significant. Enrichment in M42 is driven by ApoE. Modules are ordered by relatedness as illustrated in Fig. 1b. **b**, Module eigenprotein levels by allele dose (0, 1, 2) for the three SNPs identified as proximal mod-QTLs, separated by case status. M42 rs429358 AsymAD (0, 1) *P* = 6.9 × 10^–07^; M8 rs6940448 AsymAD (0,1) *P* = 0.003, AsymAD (0, 2) *P* = 5.5 × 10^–6^; M8 rs10458081 AsymAD (0, 1) *P* = 0.0007, AsymAD (0, 2) *P* = 6.7 × 10^–5^. M42 rs429358 *n* = 93 control (0), 10 control (1), 138 AsymAD (0), 43 AsymAD (1), 95 AD (0), 51 AD (1), 5 AD (2). M8 rs6940448 *n* = 52 control (0), 40 control (1), 11 control (2), 80 AsymAD (0), 81 AsymAD (1), 20 AsymAD (2), 60 AD (0), 70 AD (1), 21 AD (2). M8 rs10458081 n = 52 control (0), 40 control (1), 11 control (2), 77 AsymAD (0), 85 AsymAD (1), 19 AsymAD (2), 61 AD (0), 70 AD (1), 20 AD (2). **c**, M42 matrisome module eigenprotein levels by *APOE* genotype, separated by case status. AsymAD 2/2 or 2/3 to 3/4 or 4/4 *P* = 0.0002; AsymAD 3/3 to 3/4 or 4/4 *P* = 0.005; AD 2/3 to 3/4 or 4/4 *P* = 0.01. Control *n* = 20 (2/2 or 2/3), 75 (3/3), 10 (3/4 or 4/4); AsymAD *n* = 26 (2/2 or 2/3), 122 (3/3), 45 (3/4 or 4/4); AD *n* = 15 (2/3), 97 (3/3), 66 (3/4 or 4/4). **d**, ApoE levels by *APOE* genotype, separated by case status. ApoE is increased in AsymAD and AD, but *APOE* genotype does not affect ApoE levels. Control *n* = 20 (2/2 or 2/3), 75 (3/3), 10 (3/4 or 4/4); AsymAD *n* = 26 (2/2 or 2/3), 122 (3/3), 45 (3/4 or 4/4); AD *n* = 15 (2/3), 97 (3/3), 66 (3/4 or 4/4). Full statistics are provided in Supplementary Table 26. Differences in eigenprotein levels were assessed by one-way ANOVA with Tukey test. Only significant differences within case status group are shown. *P < 0.05, **P < 0.01 and ***P < 0.001. Box plots represent the median and 25th and 75th percentiles, and box hinges represent the interquartile range of the two middle quartiles within a group. Data points up to 1.5 times the interquartile range from the box hinge define the extent of whiskers (error bars). MHC, major histocompatibility complex.

We assessed which TMT AD network modules were enriched in genetic loci associated with AD as identified by genome-wide association study (GWAS) using a gene-based test of association (Fig. 4a and Supplementary Tables 23–25)^7,39,40^. We found that M42 matrisome, M30 proteasome and M29 glycosylation/ER modules were significantly enriched for AD risk genes, whereas the M7 MAPK/metabolism module demonstrated a trend toward enrichment. The strong enrichment in M42 was driven by the ApoE protein within the module, given its large effect size on AD risk^1^.

Although gene-based tests of association provide information on whether network modules are likely to be involved in upstream disease processes, they do not provide information on how variation in the genome influences module levels. To address this question, we performed a module quantitative trait loci (mod-QTL) analysis using genome-wide genotyping available from both ROSMAP and Banner cohorts. At a genome-wide level of significance and after adjusting for diagnosis and sex, among other variables, we found that rs429358 was a proximal mod-QTL (within 1 Mb—that is, *cis*) for the M42 matrisome module (Table 1). rs429358 is located in the *APOE* locus and defines the *APOE ε4* allele. This mod-QTL was further evident when we plotted the M42 eigenprotein by dose of the rs429358 single-nucleotide polymorphism (SNP) (Fig. 4b). Furthermore, M42 was the only module observed to vary by *APOE* genotype (Fig. 4c). Notably, rs429358 did not affect ApoE protein levels when tested in a linear regression model adjusting for diagnosis (*P* = 0.44). This was confirmed when we analyzed ApoE protein levels by genotype and case status (Fig. 4d and Supplementary Table 26), indicating that the change in M42 levels caused by rs429358 was independent of ApoE levels.

**Table 1:** TMT AD network mod-QTLs

SNP	CHR	BP	A1	Nearest coding gene to SNP	Beta	*P* value	Module	mod-QTL	Module protein within 1 Mb of SNP
rs28716042	8	125,418,451	G	*TMEM65*	0.04	3.7 × 10^−9^	M3 oligo/myelination	*trans*	NA
rs112028701	13	104,134,726	T	*SLC10A2*	−0.04	3.1 × 10^−8^	M22 post-synaptic density	*trans*	NA
rs11021075	11	94,940,421	T	*SESN3*	0.02	4.9 × 10^−8^	M26 complement/acute phase	*trans*	NA
rs1733609	7	81,536,375	A	*CACNA2D1*	0.02	1.6 × 10^−8^	M11 cell–ECM interaction	*trans*	NA
rs429358	19	45,411,941	C	*APOE*	0.02	3.2 × 10^−8^	M42 matrisome	*cis*	APOE
rs6940448	6	3,810,805	G	*FAM50B*	−0.02	1.4 × 10^−8^	M8 protein transport	*cis*	TUBB2A
rs1553484	6	91,507,934	A	*MAP3K7*	−0.02	6.4 × 10^−9^	M29 glycosylation/ER	*trans*	NA
rs2352535	4	114,127,905	T	*ANK2*	0.02	2.4 × 10^−8^	M27 ECM	*trans*	NA

SNPs associated with the first eigenprotein of a protein module at a genome-wide significant level (*P* < 5 × 10^−8^ using Bonferroni correction) were referred to as protein co-expression mod-QTLs. mod-QTLs located within 1 Mb of one of the module proteins were defined as proximal (*cis*) mod-QTLs; otherwise, they were categorized as distal (*trans*) mod*-*QTLs. The associations were determined using linear regression and were adjusted for cognitive diagnosis, sex, ten genetic PCs and genotyping chip. A1, allele; BP, base pair; CHR, chromosome; NA, not applicable.

In summary, we found three TMT AD network modules that were significantly enriched for AD genetic risk factors, and the level of one of these modules—M42 matrisome—was influenced by genetic variation in *APOE* independent of diagnosis or ApoE protein levels, especially in the asymptomatic stage of the disease.

### MAPK/metabolism module is associated with cognitive trajectory

**Fig. 5:**
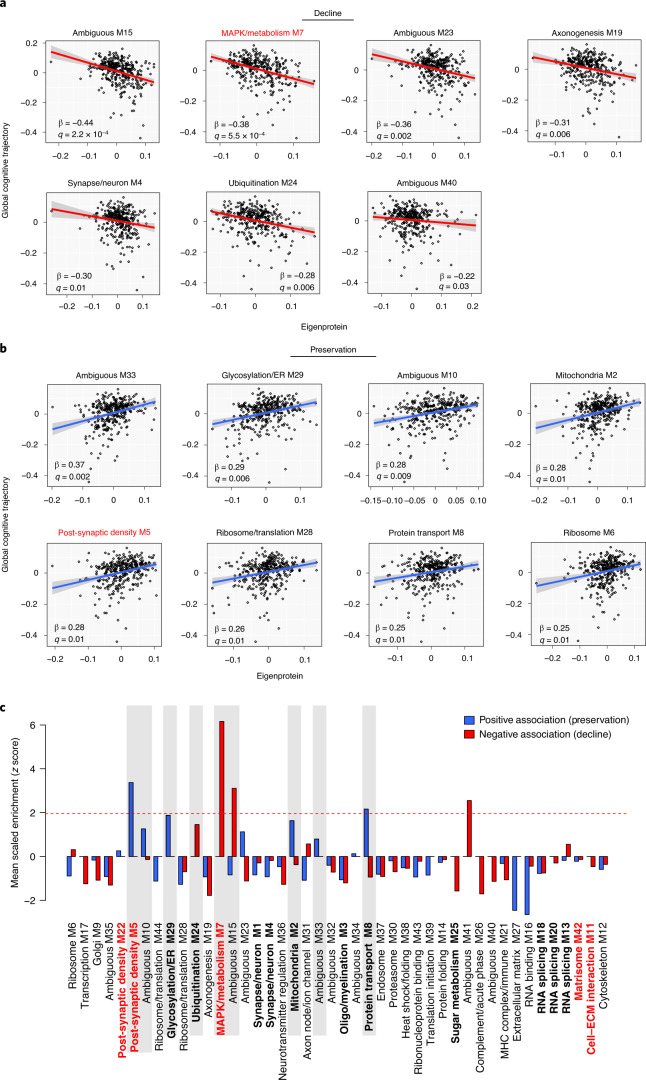
The M7 MAPK/metabolism module is associated with cognitive decline. **a**–**c**, TMT AD network modules associated with cognitive decline (**a**) or cognitive preservation (**b**) after adjustment for ten neuropathologies in ROSMAP. Eigenprotein values are plotted against the rate of cognitive change during life for each participant in ROSMAP (*n* = 328). Decline is highlighted in red; preservation is highlighted in blue. β is the effect size of module eigenprotein on cognitive trajectory after adjustment for neuropathology; *q* is the FDR significance level of this effect. Shaded areas represent 95% confidence intervals for β values. Information on the association between all TMT AD network module eigenproteins and cognitive trajectory before and after adjustment for neuropathology is provided in Supplementary Table 27. **c**, TMT AD network module enrichment of proteins positively associated with cognitive resilience (that is, preservation) or negatively associated with cognitive resilience (that is, decline) identified in a previous PWAS of cognitive resilience in the ROSMAP cohort^41^. The dashed red line indicates a *Z* score of 1.96 (*P* = 0.05), above which enrichment was considered significant. Modules that are shaded are consistent with results in **a** and **b**. Modules are ordered by relatedness as illustrated in Fig. 1b. MHC, major histocompatibility complex.

We found ten modules that were significantly associated with cognitive decline, and 11 modules that were significantly associated with cognitive preservation, without adjustment for neuropathology (Table 2 and Supplementary Table 27). Modules that were most strongly associated with cognitive decline included M7 MAPK/metabolism, M15 ambiguous and M42 matrisome, whereas modules that were strongly associated with cognitive preservation included M33 ambiguous, M5 post-synaptic density and M2 mitochondria. After adjustment for neuropathology, the M42 matrisome, M11 cell–ECM interaction, M20 RNA splicing and M25 sugar metabolism modules were no longer significantly associated with cognitive decline, and the M44 ribosome/translation, M32 ambiguous and M9 Golgi modules were no longer associated with cognitive preservation. M7 MAPK/metabolism and its related module M15 (ambiguous) remained significantly associated with rate of cognitive decline after adjustment, as well as five other modules, including the M24 ubiquitination module (Fig. 5a and Supplementary Table 27). Five of seven modules significantly associated with rate of cognitive decline were unique to the protein network. Modules that remained significantly associated with cognitive preservation after adjustment for neuropathology included the M2 mitochondria and related module M33 (ambiguous), M5 post-synaptic density and the M29 glycosylation/ER module that was unique to the protein network (Fig. 5b and Supplementary Table 27). To further examine the association of TMT AD network modules with cognitive trajectory, we assessed which modules were enriched in proteins either positively associated or negatively associated with cognitive resilience after adjustment for neuropathology, as identified by a recent proteome-wide association study (PWAS) of cognitive resilience in ROSMAP^41^ (Fig. 5c and Supplementary Tables 28 and 29). Consistent with our module association analysis, we found that M7 MAPK/metabolism and M15 ambiguous were significantly enriched in proteins negatively associated with cognitive resilience, whereas M5 post-synaptic density and M8 protein transport module were significantly enriched in proteins positively associated with cognitive resilience. Proteins identified by PWAS that were enriched in each module are provided in Supplementary Tables 28 and 29.

**Table 2:** Association of TMT AD network modules with cognitive trajectory

Module	Beta	SD_Beta	FDR *q* value	Significant after adjustment for neuropathology
Decline				
M7 MAPK/metabolism	−0.61	0.08	1.2 × 10^−10^	Yes
M15 ambiguous	−0.56	0.09	1.4 × 10^−8^	Yes
M42 matrisome	−0.49	0.09	9.3 × 10^−7^	No
M24 ubiquitination	−0.45	0.08	1.0 × 10^−6^	Yes
M23 ambiguous	−0.45	0.09	5.2 × 10^−6^	Yes
M11 cell–ECM interaction	−0.42	0.09	1.6 × 10^−5^	No
M19 axonogenesis	−0.38	0.09	8.0 × 10^−5^	Yes
M20 RNA splicing	−0.35	0.09	5.0 × 10^−4^	No
M25 sugar metabolism	−0.32	0.09	1.3 × 10^−3^	No
M4 synapse/neuron	−0.28	0.09	7.1 × 10^−3^	Yes
Preservation				
M33 ambiguous	0.51	0.09	9.8 × 10^−7^	Yes
M10 ambiguous	0.48	0.09	9.3 × 10^−7^	Yes
M5 post-synaptic density	0.47	0.09	1.5 × 10^−6^	Yes
M2 mitochondria	0.46	0.09	3.6 × 10^−6^	Yes
M29 glycosylation/ER	0.46	0.08	9.3 × 10^−7^	Yes
M28 ribosome/translation	0.42	0.09	2.0 × 10^−5^	Yes
M6 ribosome	0.36	0.09	2.5 × 10^−4^	Yes
M8 protein transport	0.35	0.09	3.0 × 10^−4^	Yes
M44 ribosome/translation	0.33	0.11	7.2 × 10^−3^	No
M32 ambiguous	0.31	0.09	2.6 × 10^−3^	No
M9 Golgi	0.27	0.09	7.1 × 10^−3^	No

The association between a module eigenprotein level for each ROSMAP participant in the TMT AD network and his or her individual cognitive trajectory was modeled with and without adjustment for neuropathology. Modules that remained significantly associated with cognitive trajectory after adjustment for neuropathology are shown in Fig. 5. Modules that had a negative association with cognitive trajectory were defined as those involved in cognitive decline, whereas modules that had a positive association were defined as those involved in cognitive preservation.

## Discussion

In this study, we analyzed more than 1,000 brain tissues by TMT-MS across multiple centers, cohorts and brain regions to develop a robust TMT AD protein network that markedly expanded upon our previous consensus LFQ AD network. Using a multilayered -omics approach, we identified new protein network modules strongly associated with AD that were not present in RNA-based networks. Some of these modules, such as the M7 MAPK/metabolism module, were associated with both AD neuropathology and cognitive trajectory in ROSMAP even after adjustment for neuropathology, and one of them—the M42 matrisome module—was influenced by the *APOE* locus. These findings highlight the utility of extending proteomic analytical depth to uncover additional AD-related protein co-expression network changes as well as the value of analyzing increasingly larger cohorts with comprehensive clinical and pathological phenotyping to provide the statistical power necessary to identify disease-relevant relationships across multi-omic datasets.

The M7 MAPK/metabolism module was strongly correlated with cognitive function and was also associated with cognitive trajectory after adjustment for neuropathology, whereas the M42 matrisome module was not associated with cognitive trajectory after adjustment for its association with neuropathology. M42 was enriched in AD genetic risk primarily due to ApoE being a member of the module, with a mod-QTL associated with the *APOE* locus. One could, therefore, postulate a model in which the pathophysiology embodied by M42 is necessary for subsequent downstream AD pathological changes, but that the pathological changes most closely associated with cognitive decline, such as those represented by the M7 MAPK/metabolism module, among others, are the prime effectors and/or modulators of cognitive decline in AD. Targeting both types of AD pathophysiology holds promise in the context of AD therapeutic development.

M42, which was not present in RNA networks, contained several proteins that have previously been identified by TMT-MS and shown to be correlated with Aβ^6,22,23,42^. These proteins, such as MDK, NTN1 and SMOC1, appear to be less reliably detected by LFQ-MS even when using LCM to isolate plaques from surrounding brain parenchyma for MS analysis, likely reflecting their lower relative abundance to other proteins within Aβ plaques or their potential weak binding affinity to plaques that may be disrupted during tissue fixation. MDK and NTN1 have previously been shown to bind directly to Aβ^22^. Interestingly, many of the M42 proteins contain heparin, heparan sulfate and glycosaminoglycan-binding domains that might mediate their interaction with Aβ plaques. ApoE, a member of the M42 module, has also been shown to interact with heparan sulfate proteoglycans, and loss of this binding interaction has been suggested as a possible mechanism for the remarkable protection afforded by the ApoE Christchurch loss-of-function mutation recently described in a presenilin-1 autosomal dominant AD mutation carrier^43,44^. Other proteins in M42 might influence Aβ plaque pathophysiology through different mechanisms, such as secreted frizzled-related protein 1 (SFRP1), which modulates Wnt signaling and has been shown to inhibit the disintegrin and metalloproteinase domain-containing protein 10 (ADAM10) that is important for regulation of Notch signaling and Aβ metabolism^33,45,46^. To what extent modulation of M42 protein levels, enzymatic activity or protein–Aβ/protein–proteoglycan interactions might affect Aβ plaque deposition or its downstream consequences remains to be determined, but such proteins represent promising therapeutic targets for AD. They might also represent promising biofluid AD biomarkers. Indeed, we have recently shown that SMOC1 is strongly elevated in AD cerebrospinal fluid^22,42,47^.

In our analysis of the association between module levels and cognitive trajectory, we observed that M7 remained associated with cognitive decline after adjustment for neuropathology, suggesting that the changes in this module in AD might not be due solely to a response to plaques and tangles. Indeed, although M7 trended toward an increase in AsymAD, the change was not significant from control. This observation, along with its association with cognitive decline, and the fact that many of the M7 hub proteins, such as UBA1, were independently associated with cognitive decline in a PWAS study of cognitive resilience^41^, would suggest that increased M7 MAPK/metabolism levels would be detrimental to cognitive function. However, robust microglial metabolic function is important for a beneficial stress response to amyloid plaques^48^ and also appears to be important for maintenance of cognitive function during aging^49^. Furthermore, M7 trended toward enrichment in AD genetic risk in this study, suggesting that, perhaps, loss of function of M7 is detrimental to cognitive function. This apparent paradox was also observed in our previous LFQ study in which increased levels of the M4 astrocyte/microglia metabolism module—the parent module of M7 and M11—were strongly associated with reduced cognitive function, yet many key M4 proteins were noted to be associated with beneficial inflammatory responses in mouse models and decreased in cases of rapidly progressive AD. M4 was also enriched in AD genetic risk. Therefore, the stress response embodied by M7 might serve both beneficial and detrimental roles in AD, and determining which aspects of a potential beneficial response to augment, or detrimental response to inhibit, will likely require direct modulation experiments in appropriate animals models or human clinical trials as well as biomarkers to measure such a response. Further study and modulation of the biological response represented by M7 represents a key goal in AD therapeutic development.

Our study demonstrates the importance of analyzing proteins directly in addition to their coding transcripts. As might be expected for a disease defined by cognitive decline in the presence of characteristic protein dysmetabolism, this observation indicates that a significant proportion of biological changes relevant to AD pathophysiology are occurring through mechanisms that are not reflected through changes in mRNA abundance or co-expression and highlights the importance of integrating multiple levels of -omics data to further understanding of the disease and selecting potential targets for disease-modifying therapeutic development.

## Methods

### Brain tissue samples and case classification

Brain tissue used in this study was obtained from the autopsy collections of the Banner Sun Health Research Institute^11^, the Mount Sinai School of Medicine Brain Bank, the ROSMAP^50^ and the Emory Alzheimer’s Disease Research Center. Tissue was from the DLPFC (BA9), frontal cortex (BA6 and BA10), anterior cingulate (BA24), temporal cortex (BA37) or PHG (BA36), as indicated. Human postmortem tissues were acquired under proper institutional review board protocols at each respective institution. Postmortem neuropathological evaluation of neuritic plaque distribution was performed according to the Consortium to Establish a Registry for Alzheimer’s Disease (CERAD) criteria^13^, and extent of spread of neurofibrillary tangle pathology was assessed with the Braak staging system^12^. Other neuropathological diagnoses were made in accordance with established criteria and guidelines^51^. All case metadata are provided in Supplementary Tables 1, 13–16 and 30. Case classification harmonization across cohorts was performed using the following rubric: cases with CERAD 0–1 and Braak 0–3 without dementia at last evaluation were defined as control (if Braak equals 3, then CERAD must equal 0); cases with CERAD 1–3 and Braak 3–6 without dementia at last evaluation were defined as AsymAD; cases with CERAD 2–3 and Braak 3–6 with dementia at last evaluation were defined as AD. Dementia was defined as MMSE < 24 or CDR ≥ 1, based on previous comparative study^52^.

### Brain tissue homogenization and protein digestion

For ROSMAP and Banner tissues, procedures were performed essentially as described^23,40^. Approximately 100 mg (wet tissue weight) of brain tissue was homogenized in 8 M urea lysis buffer (8 M urea, 10 mM Tris, 100 mM NaH_2_PO_4_, pH 8.5) with HALT protease and phosphatase inhibitor cocktail (Thermo Fisher Scientific) using a Bullet Blender (Next Advance). Each RINO sample tube (Next Advance) was supplemented with ~100 μl of stainless steel beads (0.9–2.0 mm blend, Next Advance) and 500 μl of lysis buffer. Tissues were added immediately after excision and homogenized with Bullet Blender at 4 °C with two full 5-min cycles. The lysates were transferred to new Eppendorf LoBind tubes and sonicated for three cycles consisting of 5 s of active sonication at 30% amplitude, followed by 15 s on ice. Samples were then centrifuged for 5 min at 15,000*g* and the supernatant transferred to a new tube. Protein concentration was determined by bicinchoninic acid assay (Pierce). For protein digestion, 100 μg of each sample was aliquoted, and volumes were normalized with additional lysis buffer. Samples were reduced with 1 mM dithiothreitol at room temperature for 30 min, followed by 5 mM iodoacetamide alkylation in the dark for another 30 min. Lysyl endopeptidase (Wako) at 1:100 (wt/wt) was added, and digestion was allowed to proceed overnight. Samples were then seven-fold diluted with 50 mM ammonium bicarbonate. Trypsin (Promega) was then added at 1:50 (wt/wt), and digestion was carried out for another 16 h. The peptide solutions were acidified to a final concentration of 1% (vol/vol) formic acid (FA) and 0.1% (vol/vol) trifluoroacetic acid (TFA) and de-salted with a 30-mg HLB column (Oasis). Each HLB column was first rinsed with 1 ml of methanol, washed with 1 ml of 50% (vol/vol) acetonitrile (ACN) and equilibrated with 2× 1 ml of 0.1% (vol/vol) TFA. The samples were then loaded onto the column and washed with 2× 1 ml of 0.1% (vol/vol) TFA. Elution was performed with 2 volumes of 0.5 ml of 50% (vol/vol) ACN. An equal amount of peptide from each sample was aliquoted and pooled as the pooled global internal standard (GIS), which was split and labeled in each TMT batch as described below. This was performed separately for each cohort except for the ROSMAP BA6 and BA37 tissues, which were batched together and shared a GIS at the protein level before digestion. Procedures for tissue homogenization of the Mount Sinai and Emory cohorts were performed as previously described^5,22^.

### Isobaric TMT peptide labeling

Before TMT labeling, cases were randomized by covariates (age, sex, PMI, diagnosis, etc.), into the appropriate number of batches. Peptides from each individual case and the GIS pooled standard or bridging sample (at least one per batch) were labeled using the TMT 10-plex kit (Thermo Fisher Scientific, 90406) for ROSMAP BA9 tissues and TMT 10-plex kit plus channel 11 (131C, lot no. SJ258847) for ROSMAP BA6/BA37 and Banner tissues. In each batch, up to two TMT channels were used to label GIS standards, and the remaining TMT channels were reserved for individual samples after randomization. Labeling was performed as previously described^5,6,23^. In brief, each sample (containing 100 μg of peptides) was re-suspended in 100 mM TEAB buffer (100 μl). The TMT labeling reagents (5 mg) were equilibrated to room temperature, and anhydrous ACN (256 μl) was added to each reagent channel. Each channel was gently vortexed for 5 min, and then 41 μl from each TMT channel was transferred to the peptide solutions and allowed to incubate for 1 h at room temperature. The reaction was quenched with 5% (vol/vol) hydroxylamine (8 μl) (Pierce). All channels were then combined and dried by SpeedVac (Labconco) to approximately 150 μl and diluted with 1 ml of 0.1% (vol/vol) TFA and then acidified to a final concentration of 1% (vol/vol) FA and 0.1% (vol/vol) TFA. Labeled peptides were de-salted with a 200-mg C18 Sep-Pak column (Waters). Each Sep-Pak column was activated with 3 ml of methanol, washed with 3 ml of 50% (vol/vol) ACN and equilibrated with 2× 3 ml of 0.1% TFA. The samples were then loaded, and each column was washed with 2× 3 ml of 0.1% (vol/vol) TFA, followed by 2 ml of 1% (vol/vol) FA. Elution was performed with 2 volumes of 1.5-ml 50% (vol/vol) ACN. The eluates were then dried to completeness using a SpeedVac.

For the Emory cohort, an aliquot equivalent to 20 μg was taken from each sample and combined to make one GIS per brain region. All peptide mixtures were dried under vacuum. For each tissue region, TMT 10-plex kits (Thermo Fisher Scientific) were used to label the 40 samples and ten GIS mixtures. In each batch, TMT channels 126 and 131 were used to label GIS standards, whereas the eight middle TMT channels were used to label two samples from each disease state, as previously described^23^. Labeling was performed according to the manufacturer’s protocol. In brief, each sample (containing 80 μg of peptides) was resuspended in 100 mM TEAB buffer (100 μl). The TMT labeling reagents were equilibrated to room temperature, and anhydrous acetonitrile (41 μl) was added to each reagent channel and softly vortexed for 5 min. Peptide suspensions were transferred to the corresponding TMT channels and incubated for 1 h at room temperature. The reaction was quenched with 5% (vol/vol) hydroxylamine (8 μl). To ensure complete labeling, select channels from each batch were analyzed by liquid chromatography coupled to tandem mass spectrometry (LC–MS/MS) according to previously published methods^53^. All ten channels were then combined and dried by vacuum to ~500 μl. Sep-Pak de-salting was performed, and the eluate was then dried to completeness using a SpeedVac.

For the Mount Sinai cohort, before TMT labeling the 198 samples were randomized by covariates (protein quality, sample concentration, diagnosis, age and sex) into 20 batches (ten cases per batch)^22^. The digested peptides were resuspended in 50 mM HEPES (pH 8.5) and labeled with the TMT 11-plex kit (Thermo Fisher Scientific) according to the manufacturer’s protocol. In each batch, TMT channel 126 was used to label GIS samples. All 11 channels were mixed equally and de-salted with a 100-mg C18 Sep-Pak column (Waters) for subsequent fractionation.

### High-pH off-line fractionation

High-pH fractionation was performed essentially as described^5,54^ with slight modification. Dried samples were re-suspended in high-pH loading buffer (0.07% vol/vol NH_4_OH, 0.045% vol/vol FA, 2% vol/vol ACN) and loaded onto an Agilent ZORBAX 300 Extend-C18 column (2.1 mm × 150 mm with 3.5-µm beads). An Agilent 1100 HPLC system was used to carry out the fractionation. Solvent A consisted of 0.0175% (vol/vol) NH_4_OH, 0.01125% (vol/vol) FA and 2% (vol/vol) ACN; solvent B consisted of 0.0175% (vol/vol) NH_4_OH, 0.01125% (vol/vol) FA and 90% (vol/vol) ACN. The sample elution was performed over a 58.6-min gradient with a flow rate of 0.4 ml min^−1^. The gradient consisted of 100% solvent A for 2 min, then 0–12% solvent B over 6 min, then 12–40% over 28 min, then 40–44% over 4 min, then 44–60% over 5 min and then held constant at 60% solvent B for 13.6 min. A total of 96 individual equal volume fractions were collected across the gradient and subsequently pooled by concatenation^54^ into 24 fractions and dried to completeness using a SpeedVac. Off-line fractionation of the Mount Sinai and Emory cohorts was performed as previously described^5,22^.

### TMT mass spectrometry

All fractions were resuspended in an equal volume of loading buffer (0.1% FA, 0.03% TFA, 1% ACN) and analyzed by LC–MS/MS essentially as described^55^, with slight modifications. Peptide eluents were separated on a self-packed C18 (1.9 μm, Dr. Maisch) fused silica column (25 cm × 75 μM internal diameter, New Objective) by a Dionex UltiMate 3000 RSLCnano liquid chromatography system (Thermo Fisher Scientific) for the ROSMAP samples and an Easy-nLC system (Thermo Fisher Scientific) for the Banner samples. ROSMAP peptides were monitored on an Orbitrap Fusion mass spectrometer (Thermo Fisher Scientific), and Banner peptides were monitored on an Orbitrap HF-X mass spectrometer (Thermo Fisher Scientific). For ROSMAP BA9 samples, elution was performed over a 180-min gradient with flow rate at 225 nl min^−1^. The gradient was from 3% to 7% buffer B over 5 min, then 7% to 30% over 140 min, then 30% to 60% over 5 min, then 60% to 99% over 2 min, then held constant at 99% solvent B for 8 min and then back to 1% B for an additional 20 min to equilibrate the column. Buffer A was water with 0.1% (vol/vol) FA, and buffer B was 80% (vol/vol) ACN in water with 0.1% (vol/vol) FA. For ROSMAP BA6/BA37 samples, sample elution was performed over a 120-min gradient with flow rate of 300 nl min^−1^ with buffer B ranging from 1% to 50% (buffer A: 0.1% FA in water; buffer B: 0.1% FA in 80% ACN). The mass spectrometer was set to acquire in data-dependent mode using the top speed workflow with a cycle time of 3 s. Each cycle consisted of one full scan followed by as many MS/MS (MS2) scans that could fit within the time window. For ROSMAP BA9 tissues, the full scan (MS1) was performed with an *m*/*z* range of 350–1,500 at 120,000 resolution (at 200 *m*/*z*) with AGC set at 4 × 10^5^ and maximum injection time of 50 ms. The most intense ions were selected for higher-energy collision-induced dissociation (HCD) at 38% collision energy with an isolation of 0.7 *m*/*z*, a resolution of 30,000, an AGC setting of 5 × 10^4^ and a maximum injection time of 100 ms. Five of the 50 TMT batches were run on the Orbitrap Fusion mass spectrometer using the SPS-MS3 method as previously described^23^. For ROSMAP BA6/BA37 tissues, full MS scans were collected at a resolution of 120,000 (400–1,400 *m*/*z* range, 4 × 10^5^ AGC, 50-ms maximum ion injection time). All HCD MS/MS spectra were acquired at a resolution of 60,000 (1.6 *m*/*z* isolation width, 35% collision energy, 5 × 10^4^ AGC target, 50-ms maximum ion time). Dynamic exclusion was set to exclude previously sequenced peaks for 20 s within a 10-ppm isolation window. For Banner samples, elution was performed over a 120-min gradient at a flow rate of 300 nl min^−1^ with buffer B ranging from 1% to 40% (buffer A: 0.1% FA in water; buffer B: 0.1% FA in ACN). The mass spectrometer was set to acquire data in positive ion mode using data-dependent acquisition. Each cycle consisted of one full MS scan followed by a maximum of ten MS/MS scans. Full MS scans were collected at a resolution of 120,000 (350–1,500 *m*/*z* range, 3 × 10^6^ AGC, 50-ms maximum ion injection time). All HCD MS/MS spectra were acquired at a resolution of 45,000 (0.7 *m*/*z* isolation width, 35% collision energy, 1 × 10^5^ AGC target, 96-ms maximum ion time). Dynamic exclusion was set to exclude previously sequenced peaks for 20 s within a 10-ppm isolation window. TMT mass spectrometry of the Mount Sinai and Emory cohorts was performed as previously described^5,22^.

### Database searches and protein quantification

All RAW files (1,200 RAW files generated from 50 TMT 10-plexes for ROSMAP BA9 tissues; 624 RAW files generated from 26 TMT 11-plexes for ROSMAP BA6/BA37 tissues; 528 RAW files generated from 22 TMT 11-plexes for Banner tissues; 760 RAW files generated from 19 TMT 11-plexes for Mount Sinai tissues; 210 RAW files generated from ten TMT 10-plexes for Emory tissues) were analyzed using the Proteome Discoverer suite (version 2.3, Thermo Fisher Scientific). MS2 spectra were searched against the UniProtKB human proteome database containing both Swiss-Prot and TrEMBL human reference protein sequences (90,411 target sequences downloaded on 21 April 2015), plus 245 contaminant proteins. The Sequest HT search engine was used, and parameters were specified as follows: fully tryptic specificity, maximum of two missed cleavages, minimum peptide length of 6, fixed modifications for TMT tags on lysine residues and peptide N-termini (+229.162932 Da) and carbamidomethylation of cysteine residues (+57.02146 Da), variable modifications for oxidation of methionine residues (+15.99492 Da) and deamidation of asparagine and glutamine (+0.984 Da), precursor mass tolerance of 20 ppm and a fragment mass tolerance of 0.05 Da for MS2 spectra collected in the Orbitrap (0.5 Da for the MS2 from the SPS-MS3 batches). Percolator was used to filter peptide spectral matches and peptides to a false discovery rate (FDR) of less than 1%. After spectral assignment, peptides were assembled into proteins and were further filtered based on the combined probabilities of their constituent peptides to a final FDR of 1%. Multi-consensus was performed to achieve parsimony across individual batches. In cases of redundancy, shared peptides were assigned to the protein sequence in adherence with the principles of parsimony. As default, the top matching protein or ‘master protein’ is the protein with the largest number of unique peptides and with the smallest value in the percent peptide coverage (that is, the longest protein). In cases where more than one isoform was scored equally, the additional ‘candidate master proteins’ can be queried from the .pdresult file provided on the https://www.synapse.org/DeepConsensus. Reporter ions were quantified from MS2 or MS3 scans using an integration tolerance of 20 ppm with the most confident centroid setting. Only unique and razor (that is, parsimonious) peptides were considered for quantification.

For the multi-consensus of Banner plus ROSMAP BA9 cases, peptide-specific TMT reporter abundance was first corrected within TMT batches using the ‘purityCorrect’ function of the MSnbase R package before summing of reporter abundance of parsimonious groups of peptides. The ‘purity matrix’ listing the fraction of specific reporter signal was assembled using TMT labeling reagent lot-specific information for the following batches (ROSMAP, batches 1–11: 10-plex kit lot RF234620; batches 12–50: channel-specific lots SG253447 (126), SG253458 (127N), SG255461 (127C), SF253450 (128N), SG253451 (128C), SH255464 (129N), SH255465 (129C), SF253465 (130N), SH253466 (130C) and SG253467 (131N)); all Banner batches used the same ten channel-specific lots as ROSMAP batches 12–50, plus channel 11 (131C) lot SJ258847. After correction, peptide quantitation was summed for razor plus unique peptides, thereby assembling protein abundances. Protein abundances were normalized by scaling sums of protein signal within a channel for each specific case protein sample to the maximum channel-specific protein abundance sum, as is typically calculated in the ‘normalized abundance’ columns in Proteome Discoverer output.

### Controlling for batch-specific variance across proteomics datasets

We used a tunable median polish approach termed TAMPOR to remove technical batch variance in the proteomic data, as previously described^7^. TAMPOR is a variation on the standard median polish approach^56^ to remove intra-batch, inter-batch and inter-cohort technical variance while preserving meaningful biological variance in protein expression values, normalizing to the central tendency (median) of selected intra-batch or intra-cohort samples. This approach removes batchwise technical variance in a manner preserving other variance and is robust to outliers and up to 50% missing values. The algorithm is fully documented and available as an R function, which can be downloaded from https://github.com/edammer/TAMPOR. If a protein had more than 50% missing values, it was removed from the matrix. No imputation of missing values was performed for any cohort.

Before merging the protein abundances in the consensus ROSMAP and Banner cohorts, the multi-consensus normalized protein abundance data matrix was first segregated by cohort. Intra-cohort batch effects in the Banner cohort (22 TMT 11-plex batches) and ROSMAP cohort (50 TMT 100plex batches) were then normalized separately in two steps, which were iterated until convergence. The first step transforms TMT reporter abundance into a ratio according to Eq. 1, followed by log_2_ transformation. The second step scales all sample median protein log_2_ abundance ratios to 0 and then unlogs the ratios and multiplies the ratios by median protein relative abundance factors recorded before step 1. The process is iterated until convergence, with output available as log_2_(normalized ratio) or unlogged normalized relative TMT reporter intensities.

1 $$\begin{array}{rcl}{\mathbf{Abundance}}\,{\mathbf{Ratio}}_{{\mathbf{Step}}1} & = & \frac{{sample\,reporter\,abundance}}{{median(GIS)intrabatch}} \\ && \ast \frac{{grand\,interbatch\,median(medians\left( {NON - GIS} \right)intrabatch)}}{{median\left( {\frac{{sample\,reporter\,abundance}}{{median\left( {{{{\boldsymbol{NON}}}} - GIS\,SAMPLEs} \right)intrabatch}}} \right)}}\end{array}$$

For the Banner cohort, Eq. 1 (representing TAMPOR step 1) leveraged the median protein abundance from the pooled GIS TMT channels as the denominators in both of the factors to normalize sample-specific protein abundances across the Banner cohort batches (Extended Data Fig. 1c). For the ROSMAP cohort, Eq. 1 was employed exactly as specified, using the median of non-GIS samples balanced for diagnosis and other traits across the 50 ROSMAP TMT batches and, for the second factor numerator, the grand median of all batch-specific second-term denominators. The use of non-GIS sample intra-batch medians was needed to completely adjust for batch effect and allow GIS samples to exist as outliers in the final normalized data (Extended Data Fig. 1b). After removal of intra-cohort batch effects in Banner and ROSMAP cohorts separately, all samples except cohort-specific GIS samples were processed jointly with TAMPOR into a single reassembled multi-consensus sample–protein matrix using the median of within-cohort control cases as the central tendency, enforcing that the population of all log_2_(ratio) output for control samples within the final 598 Banner plus ROSMAP samples would tend toward 0. All other cohorts were normalized essentially as described for the Banner cohort using pooled cohort GIS channels.

### Outlier removal and regression of unwanted covariates

In the consensus ROSMAP plus Banner BA9 TMT protein abundance matrix, 516 of 598 individual case samples could be classified as AD, AsymAD or control according to our classification scheme. The other 82 cases were excluded (diagnosis labeled ‘Exclude’ in traits) at this point. We removed outliers detected by network connectivity *Z*-transformed metric for a sample, using a pre-specified threshold of |Z.k | >3 standard deviations from the mean Z.k, iteratively until no further detection, as previously described using the ‘SampleNetworks’ R script (version 1.06)^7,40,57^. We then ran a pioneer round of bootstrap regression (described below) before repeating the outlier check procedure. All outliers (15 before and 13 after pioneer regression) were removed from the unregressed data, and the remaining 488 case samples were regressed the same as in the pioneer round of bootstrap regression.

The consensus ROSMAP plus Banner BA9 TMT matrix, and each of the other cohorts’ protein abundance matrices, were subjected to non-parametric bootstrap regression by subtracting the trait of interest (age at death, sex or postmortem interval (PMI)) times the median estimated coefficient from 1,000 iterations of fitting for each protein in the cohort-specific log_2_(abundance) matrix. Ages at death used for regression were uncensored. Case status/diagnosis was also explicitly modeled (that is, protected) in each regression. After regression of each individual cohort, we assessed whether any cohort-specific tissue dissection bias was present by performing a Spearman rank correlation of traits including age, sex, PMI and white matter markers to the top five PCs of log_2_(abundance). Any new outliers introduced by regression were not considered in the PCs. No gross difference in percent variance explained by any of the top five PCs with white matter correlation was observed.

### ROSMAP and Mount Sinai RNA batch correction and pre-processing

Regression of the RNA-seq data was modeled on Sieberts et al.^58,59^. Raw RNA counts were loaded, and variance partitioning was determined. Genes that were expressed at a level of more than 1 count per million (CPM) total reads in at least 50% of the samples were filtered for analysis using the CPM function edgeR (version 3.24.3). Genes were further filtered to include those with available gene length and percentage GC content from the BioMart December 2016 archive (biomaRt version 2.38.0). This left 15,582 genes and 633 samples after filtering. Samples with no RNA integrity number, PMI, sex or age at time of death were removed (*n* = 2, leaving 631 total samples). Using our diagnostic criteria, cases were again filtered to include only those in the AD (*n* = 203), AsymAD (*n* = 205) and control (*n* = 125) categories (total *n* = 533).

The raw counts were normalized in two steps. First, to account for variations in percent GC and gene length, conditional quantile normalization (CQN, version 1.28.1) was used^60^. Second, a weighted linear model was applied to the raw CPM counts using the voom-limma package (version 3.38.3) in Bioconductor (version 3.12) to estimate the confidence of sampling abundance^60,61^.

Before normalization with the voom-limma package, sample outliers were detected using principal component analysis (PCA) and the aberrant distribution of the log(CPM)^62,63^. Based on the expression pattern and the first two PCs, one sample was determined to be an outlier and removed from the data. No genes were determined to be outliers. Genes that were above and below 3 standard deviations of the aberrant distribution of the log(CPM) counts were assigned not applicable (NA) values. The final raw counts matrix before voom-limma normalization was *n* = 15,582 genes by *n* = 532 samples.

Using PCA analysis, the significant covariates in the data were determined (FDR < 0.1). Owing to the correlated nature of the covariates, it is advantageous to normalize and adjust the expression matrix using an iterative approach. This was accomplished using the voom-limma package. The primary variable of interest (diagnosis) was excluded from the pool of available covariates for selection, thereby protecting it from normalization. In each round of iteration, the residual covariates were determined from the PCA analysis and were used to construct a design matrix. Voom weights were estimated for dispersion control. A linear model was then fit to the CQN expression using the voom weights and design matrix. Using the new matrix, the PCs of the residual gene expression and a new set of significant covariates were determined. If any significant residual covariates remained with FDR < 0.1, the normalization was repeated.

### Differential expression analysis

Differentially expressed proteins were identified using one-way ANOVA followed by Holm post hoc correction of all pairwise comparisons. Significantly altered proteins with corresponding adjusted *P* value are provided in Supplementary Table 2. Differential expression is presented as volcano plots, which were generated with the ggplot2 package in R (version 3.5.2) or the matplotlib package (version 3.3.2 and version 3.3.4) in Python (version 3.8.5).

### WGCNA

We used the WGCNA algorithm (version 1.69) for our network analysis pipeline, as previously described^7,40,42^. A weighted protein co-expression network for the Banner plus ROSMAP BA9 consensus zero-centered log_2_(ratio) data was generated using the *n* = 8,826 log_2_ protein abundance × *n* = 488 case–sample matrix that had undergone reporter purity, batch effect and other covariate correction as well as network connectivity outlier (*n* = 28) removal as described above. Soft threshold power was determined for the dataset as a dataset-specific scale-free topology power based on the following two guidelines: (1) the power in a plot of power (*x*) versus R^2^ (y) should be where the R^2^ has approached an asymptote, usually near or above 0.80, and (2) the mean and median connectivity at that power should not be exceedingly high, preferably around 100 or less. The power at which these criteria are met is a tradeoff between cleaning up spurious correlations due to chance (particularly important when total samples in the network are low) and maintaining sensitivity of the clustering to still be able to pick up correlations in as much of the data as possible.

An initial network was built as described below with power = 7.0. Upon so doing, a single module of *n* = 64 proteins was found to harbor proteins assembled from mis-cleaved tryptic peptides, with higher variance in the Banner cohort driving module membership. To remove this data artifact, the clean abundance matrix values for the 64 proteins specific to measurement in Banner case samples were set to missing values, and then enforcement of the 50% missing value threshold resulted in final input for the consensus network of *n* = 8,619 proteins across *n* = 488 case samples. We confirmed that the 57 surviving proteins from the aberrant module were dispersed into diverse modules in the final network, indicating resolution of the data artifact due to this minor differential protein digestion in the Banner cohort.

The WGCNA::blockwiseModules() function was used with the following settings for the consensus network: soft threshold power = 7.0, deepSplit = 4, minimum module size of 20, merge cut height of 0.07, mean topological overlap matrix (TOM) denominator, a signed network with partitioning about medioids (PAM) respecting the dendrogram and a reassignment threshold of *P* < 0.05, with clustering completed within a single block. Specifically, this approach calculates pairwise biweight mid-correlations (bicor, a robust correlation metric) between each protein pair and transforms this correlation matrix into a signed adjacency matrix. The connection strength of components within this matrix is used to calculate a TOM, which represents measurements of protein expression pattern similarity across cohort samples constructed on the pairwise correlations for all proteins within the network. Hierarchical protein correlation clustering analysis by this approach was conducted using 1-TOM, and initial module identifications were established using dynamic tree cutting as implemented in the WGCNA::blockwiseModules() function. Module eigenproteins were defined, which are the most representative abundance value for a module equivalent to the module’s first PC, and which explain covariance of all proteins within each module^64^. Using the signedKME function in WGCNA, a table of bicor correlations between each protein and each module eigenprotein was obtained; this module membership measure is defined as k_ME_. After blockwiseModules network construction, 44 modules consisting of 18 or more proteins were detected. To enforce a kME table with no aberrant assignments to modules, a post hoc cleanup procedure was applied in which proteins with an intramodular kME less than 0.30 were removed. Then, reassignment of (1) any gray proteins (unassigned to a module) with a maximum kME to any module of more than 0.30 and (2) proteins with intramodular kME more than 0.10 below the maximum kME of the protein’s correlation to any other module was done to reassign each such protein to the module corresponding to the protein’s maximum kME. Then, MEs and the signed kME table were recalculated with the WGCNA::moduleEigengenes() and WGCNA::signedKME() functions, respectively. Finally, the kME table individual protein reassignment process was repeated if additional corrections could be made, up to a total of 30 iterations. For the consensus network, this required 11 iterations until resolution, which increased the module size of the smallest module (M44) in the network to 28, and decreased gray (unassigned) protein count for the network from 3,156 (35.8%) to 2,282 (25.9%).

The WGCNA::blockwiseModules() fucntion was also used to generate the Mount Sinai RNA network, the ROSMAP RNA network and the ROSMAP RNA protein overlap network. The parameters used to build these networks were the same as those used in the consensus network build, with the exception of the soft threshold power, which was 10.0, 12.5, 10.0 and 8.0 for the Mount Sinai RNA network, ROSMAP RNA network, ROSMAP RNA overlap network and ROSMAP protein overlap network, respectively. As in the consensus network, a post hoc kME table cleanup was applied to each network. The Mount Sinai RNA network contained 93 modules with a minimum module size of 45 genes. The ROSMAP RNA networks were similar in size, with 88 modules and a minimum module size of 49 for the *n* = 532 network and 91 modules with a minimum module size of 44 for the *n* = 168 network. The ROSMAP RNA protein overlap network contained 69 modules with a minimum module size of 13.

### MONET M1 analysis

The three top-performing methods from the Disease Module Identification DREAM Challenge were compiled in the MONET toolbox and released to the public for use (https://github.com/BergmannLab/MONET.git)^19^. We selected the M1 method from this toolbox as a complementary network analysis method to explore the AD TMT network. Unlike WGNCA’s hierarchical clustering method, the M1 method determines modules and communities by optimizing the well-known modularity function from Newman and Girvan^65^. However, unlike traditional modularity optimization methods, it searches the network at multiple topological scales, resulting in a multi-resolution approach. The authors added the resistance parameter, *r*, which averts genes from joining modules. If *r* = 0, the method returns to Newman and Girvan’s original modularity optimization; *r* > 0 produces smaller modules (or reveals network substructure); and *r* < 0 produces larger modules (or results in network superstructure)^66^. Instead of manually choosing the parameter *r*, users are allowed to optimize their network by tuning four hyperparameters: minimum module size, maximum module size, desired average degree and desired average degree tolerance. The MONET M1 algorithm will then fit a resistance value to the data to produce a network described by the user’s parameters. Input for M1 was an edge list, obtained from a cleaned abundance matrix as follows: power for scale-free toplogy was determined as described in the above WGCNA methods section for each M1 input network, and the adjacency was calculated for the clean abundance data matrix raised to this power using the WGCNA adjacency function with additional parameters Type=‘signed’, corFnc=‘bicor’ and the corOptions parameter set to use pairwise complete correlation. As M1 takes an edge list as input, the adjacency upper triangle correlation values were used to populate the weights of unique pairwise correlations in the edge list (NumPy version 1.20.1 and SciPy version 1.61). No sparsification of the edge list was applied before running M1, and neither TOM nor 1-TOM (dissimilarity) were considered.

We optimized the hyperparameters using a grid search by varying minimum module size, $i \in \left\{ {3,10,15,20} \right\}$, maximum module size, $j \in \left\{ {100,200,300,400,500} \right\}$, and desired average degree, $k \in \left\{ {25,50,75} \right\}$. The desired average degree tolerance was left at the default value of 0.2. Here, the optimal model was defined as the set of parameters that minimized the percentage of proteins not assigned to a module (Pandas version 1.2.3). The final parameters selected were $i = 3,j = 100,k = 75$, which built a network with 373 modules and 26.91% proteins not assigned to a module. After the network was built, the smallest modules were pruned from the graph so that the smallest module contained no less than 20 members in concordance with the WGCNA network (networkX version 2.5). This increased the percent of proteins not assigned to a module to 30.22% and decreased the number of modules to 87. This final network was used in module preservation studies with the network built using the WGCNA algorithm.

### Network preservation

We used the WGCNA::modulePreservation() function to assess network module preservation across cohorts. We also used this function to assess the effect of missing values on the consensus network. *Z*_summary_ composite preservation scores were obtained using the consensus network as the template versus each other cohort or missing value threshold tested, with 500 permutations. Random seed was set to 1 for reproducibility, and the quickCor option was set to 0. We also assessed network module preservation using synthetic eigenproteins. In brief, protein module members in the consensus network template with a k_ME.intramodule_ among the top 20th percentile were assembled into a synthetic module in each target cohort, and synthetic modules with at least four members were used to calculate synthetic weighted eigengenes representing the variance of all members in the target network across case samples via the WGCNA::moduleEigengenes() function. Statistics and correlation scatter plots involving target cohort traits were then calculated and visualized.

### Network module overlap and percent novelty analyses

Module membership by gene symbol was overlapped for all pairwise comparisons of modules in the current TMT consensus network (44 modules, this study) to those of the LFQ consensus network (13 modules) previously published^7^. A one-tailed Fisher exact test looking for significant overrepresentation or overlap was employed, and *P* values were corrected for multiple testing using the Benjamini–Hochberg method. In addition, novel gene products in the TMT network were identified and checked for significant overrepresentation (one tailed) in the TMT consensus 44 modules not including gray and, in a second analysis, considering only the top 20% of gene product members of modules as ranked by kME_intramodule_. Finally, one-tailed Fisher exact tests were also employed to determine module-wise overrepresentation of amyloid plaque-associated^30^ and neurofibrillary tangle-associated^31^ proteins identified in previous studies. R functions fisher.test() and p.adjust() were used to obtain the above statistics.

### GO and cell type marker enrichment analyses

To characterize differentially expressed proteins and co-expressed proteins based on GO annotation, we used GO Elite (version 1.2.5) as previously published^40,67^, with pruned output visualized using an in-house R script. Cell type enrichment was also investigated as previously published^40,67^. For the cell type enrichment analyses, we generated an in-house marker list combining previously published cell type marker lists from Sharma et al.^68^ and Zhang et al.^69^ (Supplementary Table 7). For each of the five cell types of interest (endothelia, microglia, astrocyte, neuron and oligodendrocyte), genes from the Sharma et al. list and genes from the Zhang et al. list were joined into one list per cell type. If, after the lists were merged, a gene symbol was assigned to two cell types, we defaulted to the cell type defined by the Zhang et al. list such that each gene symbol was affiliated with only one cell type. The gene symbols were then processed through MyGene to update them to the most current nomenclature and converted to human symbols using homology lookup. Fisher exact tests were performed using the human cell type marker lists to determine cell type enrichment and were corrected by the Benjamini–Hochberg procedure.

### GWAS module association

To determine if any protein products of GWAS targets were enriched in a particular module, we used the SNP summary statistics from Kunkle et al.^70^ to calculate the gene-level association value using MAGMA (version 1.08b)^39^, as previously described^40^. To remove SNPs in linkage disequilibrium with the *APOE* locus from consideration in the analysis, we excluded SNPs within a 2-Mb window centered on *APOE. APOE* was manually added to the gene list and assigned a –log *P* value of 50, given its known strong association with AD. SNPs associated with non-protein-coding genes based on information in the current version of Ensembl BioMart were also removed from consideration (*n* = 1,151). A total of 31 genes with MAGMA P_MULTI<0.05 were excluded from the analysis. A final list of 1,822 genes with gene-based GWAS *P* < 0.05, including *APOE*, was used for enrichment analysis. Similar analyses were performed with GWAS candidates for schizophrenia (SCZ) and autism spectrum disorder (ASD). These GWAS datasets were provided and downloaded from the Psychiatric Genomics Consortium (http://www.med.unc.edu/pgc/downloads).

### PWAS module association

Proteins (*n* = 8,356) tested in the PWAS study by Yu et al.^41^ for correlation to cognitive resilience (or decline, when negatively correlated) were split into lists of unique gene symbols representing protein gene products positively correlated (*n* = 645) and negatively correlated (*n* = 575) to cognitive resilience, and then these lists with corresponding *P* values were separately checked for enrichment in consensus TMT network modules using a permutation-based test (10,000 permutations) implemented in R with exact *P* values for the permutation tests calculated using the permp function of the statmod package. Module-specific mean *P* values for risk enrichment were determined as a *Z* score, specifically as the difference in mean *P* value of gene product proteins hitting a module at the level of gene symbol minus the mean *P* value of genes hit in the 10,000 random replacement permutations, divided by the standard deviation of *P* value means also determined in the random permutations. This method is identical to that used for determining module-wise enrichment of risk in GWAS results summarized as gene-level *P* values using MAGMA (see ‘GWAS module association’ section).

### Network mod-QTL analysis

DNA from ROSMAP participants underwent whole-genome sequencing (WGS) or genome-wide genotyping using either the Affymetrix GeneChip 6.0 or the Illumina OmniQuad Express chip as previously described^71^. We used WGS when multiple data sources were available. Participants from Banner were genotyped using the Affymetrix Precision Medicine Array. Quality control of WGS and array-based genotypes were performed separately using Plink (version 1.9) as described previously^72^. In brief, variants with Hardy–Weinberg equilibrium *P* < 10^−7^, with missing genotype rate greater than 5%, with minor allele frequency less than 1% and are not SNPs were removed. KING (version 2.2.2) was used to remove individuals estimated to be closer than second-degree kinship^73^. Genotypes were imputed to the 1000 Genomes Project Phase 3 (ref. ^74^) using the Michigan Imputation Server^75^. SNPs with imputation *R*^2^ > 0.3 were retained for analysis. Genetic variants associated with a protein co-expression module were identified using linear regression to model the first eigenprotein of the protein module as a function of genotype, adjusting for sex, cognitive diagnosis, ten PCs and genotyping chip. Among genetic variants with genome-wide level of significant association with a module (*P* < 5 × 10^−8^), we categorized them as either proximal or distal protein mod-QTL. Proximal mod-QTL was defined as SNPs within 1 Mb of any of the genes in the corresponding module; otherwise, they were categorized as distal mod-QTLs. mod-pQTLs were clumped by Plink using default parameters so that SNPs within 250 kb of one another and in linkage disequilibrium (r^2^ > 0.5) were represented by the lead SNP (that is, the most statistically significant SNP in the clumped locus). Association between ApoE protein levels and the rs429358 genotype was tested in a linear regression model adjusting for diagnosis, ten population PCs and cohort.

### Cognitive trajectory analysis

ROSMAP participants underwent cognitive testing annually in the domains of episodic memory, perceptual orientation, perceptual speed, semantic memory and working memory as described in detail previously^76^. The raw score from each of these 17 cognitive tests was converted to a Z score using the mean and standard deviation of the cohorts at the baseline visit. Then, the Z scores were averaged to create a composite annual global cognitive score. The rate of cognitive change over time for each participant was represented by the random slope of a linear mixed model where the annual global cognitive score was the longitudinal outcome and follow-up year was the independent variable, adjusting for age at recruitment, sex and years of education as previously described^71^. We used the person-specific random slope to represent the rate of change of cognitive performance over time for each participant. To examine associations between protein co-expression modules and cognitive trajectory, we performed linear regression with cognitive trajectory as the outcome and the first module eigenprotein as the predictor with or without adjusting for the ten measured pathologies. The ten age-related pathologies measured in ROSMAP included amyloid-β, tangles, cerebral amyloid angiopathy, cerebral atherosclerosis, arteriolosclerosis, Lewy body, TDP-43, gross infarct, microinfarct and hippocampal sclerosis as described in detail previously^77^. Multiple testing adjustment (for multiple modules) was addressed with Benjamini–Hochberg FDR^78^.

### Immunohistochemistry

Human forebrain 8-µm-thick sections were deparaffinized and processed for immunohistochemical labeling with antibodies on a Thermo Fisher Scientific autostainer. Primary antibody was rabbit anti-Midkine EP1143Y (Abcam). Secondary antibody was biotinylated goat anti-rabbit 111-065-003 (Jackson ImmunoResearch Laboratories). Sections were blocked with normal serum and incubated with primary antibody (1:1,000) and then exposed to secondary antibody (1:200) followed by avidin-biotin complex (Vector ABC Elite Kit) and developed with diaminobenzidine. After sections were mounted and coverslipped, images were captured using an Olympus bright-field microscope and camera (BX51). For final output, images were processed using Adobe Photoshop software.

### Other statistics

No statistical methods were used to predetermine sample sizes, but our sample sizes are similar to those reported previously^7^. Data collection and analysis were not performed blinded to the conditions of the experiments. All statistical analyses were performed in R (version 3.5.2). Data distribution was assumed to be normal, but this was not formally tested. Box plots represent the median and the 25th and 75th percentile extremes; thus, the hinges of a box represent the interquartile range of the two middle quartiles of data within a group. The farthest data points up to 1.5 times the interquartile range away from the box hinges define the extent of whiskers (error bars). Correlations were performed using the biweight midcorrelation function as implemented in the WGCNA R package. Comparisons between two groups were performed by *t-*test. Comparisons among three or more groups were performed with one-way ANOVA with Tukey or post hoc pairwise comparison of significance. Comparison of variance was performed using *F* test. *P* values were adjusted for multiple comparisons by FDR correction where indicated. Module membership graphs were generated using the networkX package (version 2.5) and the rpy2 package (version 3.4.3) in Python (version 3.8.5) and in-house graphing scripts.

### Reporting Summary

Further information on research design is available in the Nature Research Reporting Summary linked to this article.

## Online content

Any methods, additional references, Nature Research reporting summaries, source data, extended data, supplementary information, acknowledgements, peer review information; details of author contributions and competing interests; and statements of data and code availability are available at 10.1038/s41593-021-00999-y.

## Supplementary information

Supplementary Information Supplementary Data 1–3

Reporting Summary

Supplementary Tables

## Data Availability

Additional results and discussion from this study are available at 10.1101/2021.04.05.438450. Raw data, case traits and analyses related to this manuscript are available at https://www.synapse.org/DeepConsensus. The results published here are in whole or in part based on data obtained from the AMP-AD Knowledge Portal (https://adknowledgeportal.synapse.org). The AMP-AD Knowledge Portal is a platform for accessing data, analyses and tools generated by the AMP-AD Target Discovery Program and other programs supported by the National Institute on Aging to enable open-science practices and accelerate translational learning. The data, analyses and tools are shared early in the research cycle without a publication embargo on secondary use. Data are available for general research use according to the following requirements for data access and data attribution (https://adknowledgeportal.synapse.org/#/DataAccess/Instructions). ROSMAP resources can be requested at www.radc.rush.edu. The UniProtKB human proteome database, containing both Swiss-Prot and TrEMBL human reference protein sequences downloaded 21 April 2015, was used to search mass spectra and is available at https://ftp.uniprot.org/pub/databases/uniprot/previous_releases/.

## Code availability

The algorithm used for batch correction is fully documented and available as an R function, which can be downloaded from https://github.com/edammer/TAMPOR.
