# Supplementary material for: Large-scale deep multi-layer analysis of Alzheimer’s disease brain reveals strong proteomic disease-related changes not observed at the RNA level
Source: Nat Neurosci. 2022 Feb 3;25(2):213–25. doi: 10.1038/s41593-021-00999-y (PMC8825285; doi:10.1038/s41593-021-00999-y)
Supplement: Supplementary file 2 — Reporting Summary [file 41593_2021_999_MOESM2_ESM.pdf]

nature research

Corresponding author(s): Erik C.B. Johnson, Allan I. Levey, Nicholas T. Seyfried

Last updated by author(s): 11-15-2021

Reporting Summary

Nature Research wishes to improve the reproducibility of the work that we publish. This form provides structure for consistency and transparency in reporting. For further information on Nature Research policies, see our Editorial Policies and the Editorial Policy Checklist.

Statistics

For all statistical analyses, confirm that the following items are present in the figure legend, table legend, main text, or Methods section.

n/a	Confirmed	
☐	☒	The exact sample size (*n*) for each experimental group/condition, given as a discrete number and unit of measurement
☐	☒	A statement on whether measurements were taken from distinct samples or whether the same sample was measured repeatedly
☐	☒	The statistical test(s) used AND whether they are one- or two-sided<br>*Only common tests should be described solely by name; describe more complex techniques in the Methods section.*
☐	☒	A description of all covariates tested
☐	☒	A description of any assumptions or corrections, such as tests of normality and adjustment for multiple comparisons
☐	☒	A full description of the statistical parameters including central tendency (e.g. means) or other basic estimates (e.g. regression coefficient) AND variation (e.g. standard deviation) or associated estimates of uncertainty (e.g. confidence intervals)
☐	☒	For null hypothesis testing, the test statistic (e.g. *F*, *t*, *r*) with confidence intervals, effect sizes, degrees of freedom and *P* value noted<br>*Give P values as exact values whenever suitable.*
☒	☐	For Bayesian analysis, information on the choice of priors and Markov chain Monte Carlo settings
☒	☐	For hierarchical and complex designs, identification of the appropriate level for tests and full reporting of outcomes
☐	☒	Estimates of effect sizes (e.g. Cohen's *d*, Pearson's *r*), indicating how they were calculated

Our web collection on statistics for biologists contains articles on many of the points above.

Software and code

Policy information about availability of computer code

Data collection	Proteome Discoverer Suite (version 2.3)
Data analysis	TAMPOR batch correction (https://github.com/edammer/TAMPOR), Oldham's 'SampleNetworks' v1.06 R script for network connectivity outlier removal, Weighted Correlation Network Analysis (WGCNA) algorithm (v1.69), MONET M1 algorithm (https://github.com/BergmannLab/MONET.git), GO Elite v1.2.5, MAGMA v1.08b, R v3.5.2, Python v3.8.5, Limma v3.38.3, edgeR v3.24.3, biomaRt v2.38.0, cqn v1.28.1, rpy2 v3.4.3, networkX v2.5, SciPy v1.6.1, NumPy v1.20.1, Pandas v1.2.3, Matplotlib v3.3.2 and v3.3.4, Bioconductor v3.12, Plink v1.9, KING v2.2.2

For manuscripts utilizing custom algorithms or software that are central to the research but not yet described in published literature, software must be made available to editors and reviewers. We strongly encourage code deposition in a community repository (e.g. GitHub). See the Nature Research guidelines for submitting code & software for further information.

Data

Policy information about availability of data

All manuscripts must include a data availability statement. This statement should provide the following information, where applicable:

- Accession codes, unique identifiers, or web links for publicly available datasets
- A list of figures that have associated raw data
- A description of any restrictions on data availability

Raw data, case traits, and analyses related to this manuscript are available at https://www.synapse.org/DeepConsensus.
The UniProtKB human proteome database containing both Swiss-Prot and TrEMBL human reference protein sequences downloaded April 21, 2015 was used to search mass spectra, and is available at https://ftp.uniprot.org/pub/databases/uniprot/previous_releases/.

Field-specific reporting

Please select the one below that is the best fit for your research. If you are not sure, read the appropriate sections before making your selection.

☒ Life sciences ☐ Behavioural & social sciences ☐ Ecological, evolutionary & environmental sciences

For a reference copy of the document with all sections, see nature.com/documents/nr-reporting-summary-flat.pdf

Life sciences study design

All studies must disclose on these points even when the disclosure is negative.

Sample size	No sample size calculation was performed. Sample size was chosen based on the available data.
Data exclusions	Network outliers were removed using Oldham's 'SampleNetworks' v1.06 R script using a pre-established 3 fold-SD cutoff of Z-transformed sample connectivity. RNA sample outliers were detected using principal component analysis (PCA) and the aberrant distribution of the log(CPM).
Replication	The consensus network was replicated in ROSMAP, Mt. Sinai, and Emory cohorts. A majority of modules replicated in each cohort and brain region. All discovery and replication data are provided.
Randomization	For each cohort, samples were randomized prior to mass spectrometry-based proteomic analysis based on available traits.
Blinding	Blinding was not relevant to this study because intervention bias was not an issue given the nature of the study

Reporting for specific materials, systems and methods

We require information from authors about some types of materials, experimental systems and methods used in many studies. Here, indicate whether each material, system or method listed is relevant to your study. If you are not sure if a list item applies to your research, read the appropriate section before selecting a response.

Materials & experimental systems

n/a	Involved in the study	
☐	☒	Antibodies
☒	☐	Eukaryotic cell lines
☒	☐	Palaeontology and archaeology
☒	☐	Animals and other organisms
☒	☐	Human research participants
☒	☐	Clinical data
☒	☐	Dual use research of concern

Methods

n/a	Involved in the study	
☒	☐	ChIP-seq
☒	☐	Flow cytometry
☒	☐	MRI-based neuroimaging

Antibodies

Antibodies used	rabbit anti-Midkine EP1143Y (Abcam), biotinylated goat anti-rabbit 111-065-003 (Jackson Immunoresearch Labs)
Validation	rabbit anti-Midkine EP1143Y (Abcam) has been validated for IHC by knockout studies
